# Cytogenotoxic Effects and Physicochemical and Molecular Profiles of Eugenol‐Derived Triazoles With Phytotoxic Potential

**DOI:** 10.1002/cbdv.202503621

**Published:** 2026-01-28

**Authors:** Thayllon de Assis Alves, Thammyres de Assis Alves, Poliana Aparecida Rodrigues Gazolla, Ângela Maria Almeida Lima, Mariana Belizario de Oliveira, William dos Santos Belarmino, Camila Luiz Sena, Róbson Ricardo Teixeira, Elias Terra Werner, Othon Souto Campos, Adilson Vidal Costa, Milene Miranda Praça‐Fontes

**Affiliations:** ^1^ Departamento De Biologia Universidade Federal do Espírito Santo, Alto Universitário Alegre Espírito Santo Brazil; ^2^ Departamento De Química e Física Universidade Federal do Espírito Santo, Alto Universitário Alegre Espírito Santo Brazil; ^3^ Departamento De Química Universidade Federal de Viçosa Viçosa Minas Gerais Brazil

**Keywords:** click reaction, cytotoxicity, lettuce, mutagenesis, phytotoxicity

## Abstract

Eight eugenol‐derived 1,2,3‐triazole derivatives (**2a–2h**) were evaluated for their phytotoxic and cytogenotoxic activities. Bioassays were performed using *Lactuca sativa* to assess macroscopic parameters, including germination percentage, germination speed index, and root growth (RG), as well as microscopic parameters, such as the mitotic index (MI) and chromosomal alteration (CA) and nuclear alteration (NA). Compounds **2b** and **2e**, bearing *ortho*‐bromine and *meta*‐chlorine substituents, respectively, exhibited the highest phytotoxic activity, with effects comparable to glyphosate. In contrast, **2c**, **2d**, **2f**, **2g**, and **2h**, which contain other halogens and different aromatic patterns, stimulated RG. Cytogenetic analysis revealed that **2b** and **2e** significantly reduced the MI and increased NA and CA, indicating cytotoxic and genotoxic potential. Theoretical calculations of physicochemical properties showed that **2b**, **2e**, and **2g** are hydrophilic. In contrast, the others are lipophilic, a feature that may influence their interaction with plant tissues and help explain the observed differences. Compared with glyphosate, the compounds **2b** and **2e** exhibited a variety of intermolecular interactions due to their hydrophilic behavior, resulting in lower docking energy and surpassing the quality and quantity of interactions observed with glyphosate itself. These findings highlight **2b** and **2e** as promising candidates for the development of herbicides with alternative modes of action.

## Graphical abstract

The study evaluates the phyto and cytogenotoxic activity of eight eugenol‐derived triazole compounds in Lactuca sativa. Compounds 2b and 2e exhibit high phytotoxicity, comparable to glyphosate, as well as cytotoxic and genotoxic effects associated with their greater hydrophilicity, highlighting them as promising candidates for the development of herbicides with new modes of action.

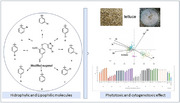

## 1 Introduction

Brazilian agricultural activities hold substantial national and international relevance, mainly due to the country's high export rates of cultivated products. According to the Brazilian Ministry of Agriculture and Livestock, the growth in agricultural exports observed in 2024 was supported by increased grain production during the 2023/2024 harvest season, which reached 297 million tons. The main crops produced were corn and soybeans, accounting for nearly 115 and 147 million tons, respectively [1].

Maintaining such high productivity levels depends heavily on the implementation of technological packages developed since the Green Revolution, which began in Brazil in the 1970s, particularly amid increasing global food demand. Among these technologies, the use of synthetic herbicidal molecules for weed control stands out as a key factor in modern agricultural systems [2].

Weeds represent a significant challenge to agricultural production, as their rapid growth often allows them to emerge before cultivated crops, leading to intense competition for essential resources such as water, nutrients, and light. This early establishment provides weeds with a competitive advantage that can ultimately result in significant yield losses [3].

Currently, synthetic herbicides are widely used due to their high cost‐benefit ratio. However, their excessive and continuous use has been associated with adverse effects on both human health and the environment [4]. In addition, changes in cropping systems and agricultural practices have contributed to the emergence of herbicide‐resistant weed species. This increasing resistance is now considered one of the most serious threats to sustainable food production systems worldwide [5, 6].

To address the growing challenge posed by herbicide‐resistant weeds, the synthesis and development of new herbicides with alternative mechanisms of action are necessary. However, the introduction of new herbicidal compounds with novel modes of action has been remarkably limited over the past four decades, further underscoring the urgency of this issue [7].

With advances in research and genetic improvement strategies, new chemical compounds are being developed to combat invasive plant species in agricultural environments. These compounds may be entirely synthetic, derived from natural products, or produced as byproducts of other production sectors [8].

The development of new approaches involving a broader diversity of agrochemicals for the control of invasive agents in agriculture has therefore emerged as a central objective of contemporary research. In this context, the exploration of natural products obtained from plants and microorganisms has gained increasing attention, as these compounds represent promising sources of new bioactive molecules [9].

Among natural products, some can be used directly as bioactive agents, whereas others can be chemically modified to generate derivatives with improved properties. A notable example is eugenol, a naturally occurring compound found in the essential oils of plants belonging to the *Lamiaceae*, *Lauraceae*, *Myrtaceae*, and *Myristicaceae* families [9].

Eugenol is widely employed as a fragrance and flavoring agent in cosmetic and food products [9]. Its biological relevance is supported by a range of well‐documented activities, including antioxidant, anti‐inflammatory, antispasmodic, antidepressant, antigenotoxic, and anticarcinogenic [10, 11]. From a chemical standpoint, the presence of an aromatic ring, a hydroxyl group, and an allyl moiety renders this molecule amenable to structural modifications, thereby enabling the synthesis of novel derivative compounds [12].

Among the promising classes of compounds for the development of new agrochemicals, triazoles deserve particular attention. These compounds are characterized by a 1,2,3‐ or 1,2,4‐triazole heterocyclic ring and are well known for their broad‐spectrum antifungal activity, primarily due to inhibition of the enzyme lanosterol 14α‐demethylase [13].

Considering the potential of natural molecules and their derivatives, such as eugenol‐based triazoles, it is essential to adopt efficient strategies to evaluate both their biological activity and safety. In this context, the use of model plant species in preliminary phytotoxicity bioassays is a fundamental tool for early‐stage herbicide discovery. Lettuce (*Lactuca sativa*), in particular, stands out as a model species due to its high sensitivity to chemical stimuli, rapid germination, and easily observable growth parameters, making it suitable for detecting the phytotoxic effects of new compounds [14, 15].

These bioassays enable rapid screening of chemical libraries to identify promising candidates that inhibit seed germination or seedling growth, thereby serving as prototypes for the development of selective and effective herbicides. They permit evaluation of macroscopic physiological parameters, such as germination rate and early seedling growth (root and shoot elongation), as well as microscopic cellular parameters. The latter include alterations in the cell division process, such as changes in the mitotic index (MI), chromosomal abnormalities, and nuclear alterations (NAs) [8, 16]. Moreover, these assays are cost‐effective, reproducible, and aligned with environmentally responsible practices, as they facilitate the early identification of compounds with undesirable toxicological profiles [17]. Consequently, model plant bioassays play a crucial role in guiding the rational design of new herbicidal agents with improved efficacy and safety.

On the basis of these premises, the present study reports the screening of a library of eugenol‐derived 1,2,3‐triazoles for their phytotoxicity on the early development of *L. sativa* to identify compounds with potential for further investigation in herbicide development. Furthermore, the physicochemical and molecular parameters of the compounds were investigated, and a principal component analysis (PCA) was performed to support the interpretation of the results.

## 2 Results and Discussion

### 2.1 Preparation of Eugenol Derivatives **2a**–**2h**

**FIGURE 1:**
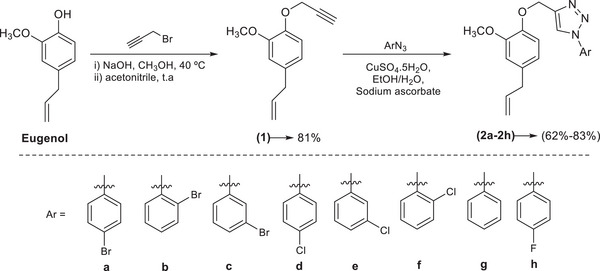
Reactions involved in the synthesis of triazole compounds **2a–2h**.

The initial step in the synthesis of the triazolic compounds **2a–2h** involved the alkylation of eugenol, yielding compound **1** in 81% yield. After obtaining compound **1**, the synthesis of eugenol‐derived triazole compounds was performed via a copper(I)‐catalyzed azide‐alkyne cycloaddition (CuAAC) reaction, commonly referred to as the click reaction. This process involved the reaction of compound **1** with different aromatic azides, affording the target products in yields ranging from 62% to 83%, as illustrated in Figure 1.

All compounds **2a–2h** were fully characterized by ^1^H and ^13^C nuclear magnetic resonance (NMR) spectroscopy, infrared (IR) spectroscopy, and mass spectrometry. The IR spectra exhibited the expected absorption bands corresponding to the functional groups present in the molecules. Notably, an absorption band observed in the region between 1588 and 1493 cm^−1^ was attributed to N═N stretching vibrations, which are characteristic of structures containing the 1,2,3‐triazole ring. In the ^1^H NMR spectra, a singlet in the range of 8.07–8.99 ppm was assigned to the hydrogen atom on the triazole ring. Additionally, the signal corresponding to the methylene hydrogens bridging the triazole ring and the oxygen atom appeared as a singlet in the range of 5.14–5.38 ppm. In the ^13^C NMR spectra, both the number of signals and their chemical shift values were consistent with the proposed molecular structures. Signals attributed to the triazolic ring carbons were observed between 120.6 and 146.8 ppm. Finally, the molecular formulas of the triazole derivatives were confirmed by LC–MS.

Following their synthesis and structural characterization, the compounds were subjected to biological assays to evaluate their phytotoxic and cytogenotoxic activities.

### 2.2 Biological Results

**FIGURE 2:**
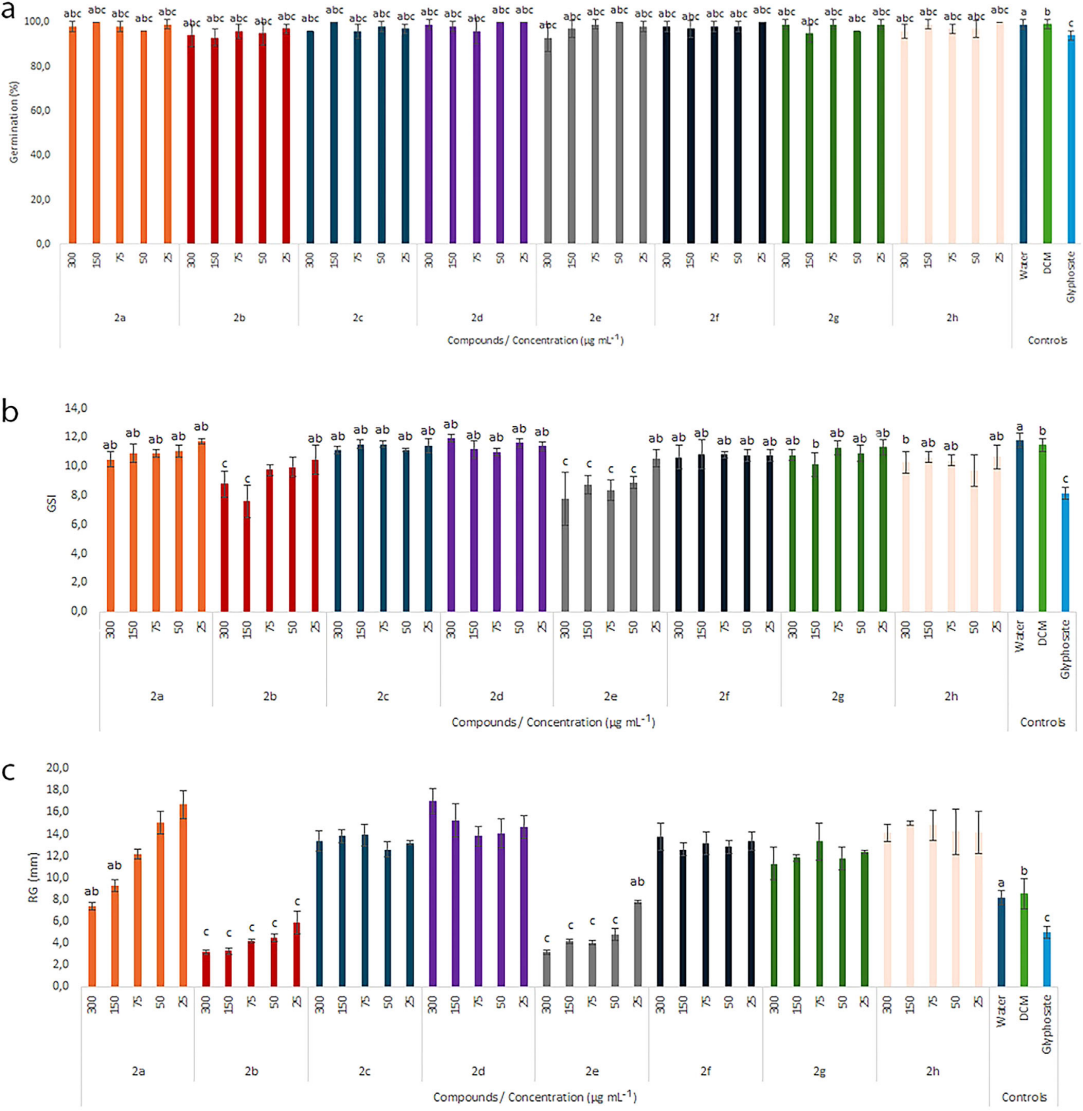
Germination percentage (a), germination speed index (GSI) (b), and root growth (RG) (mm) (c) of *Lactuca sativa* seeds (*n* = 100) after 48 h of exposure to triazole compounds **2a–2h**. Means followed by the same letter indicate no significant difference from the respective control groups, and means followed by the absence of letters indicate a difference from all controls, according to Dunnett's test (*p* value < 0.05): (a) negative control (water); (b) negative control (dichloromethane—DCM); (c) positive control (glyphosate).

The evaluated macroscopic germination parameters were the total germination percentage (GP) after 48 h of exposure to the test compounds and the germination speed index (GSI). No significant differences in GP were observed between any of the triazole treatments (**2a–2h**) and the control groups (water, dichloromethane [DCM], and glyphosate), indicating that none of the compounds tested affected *L. sativa* seed germination under the conditions evaluated (Figure 2a).

Significant inhibition of the GSI was observed at higher concentrations of compounds **2b** (300 and 150 µg mL^−1^) and for **2e** at 300, 150, 75, and 50 µg mL^−1^ (Figure 2b). These treatments exhibited GSI values statistically similar to the positive control (glyphosate) and significantly lower than those of the negative controls (water and DCM) (Figure 2b).

The germination process is preceded by the breaking of seed dormancy, which begins with increased hydration, triggering a cascade of events. This cascade naturally promotes the generation of reactive oxygen species (ROS), requiring efficient performance of the plant's anti‐stress machinery [18]. Despite the increased ROS generation, GP is the least responsive variable in toxicity tests, with changes becoming evident only under conditions of high test‐agent toxicity [19, 20].

Root growth (RG) of *L. sativa* seedlings was significantly affected by the tested eugenol‐derived 1,2,3‐triazoles (Figure 2c). Compounds **2c**, **2d**, **2f**, **2g**, and **2h** significantly stimulated RG at different concentrations, with increases ranging from 38% (**2g** at 300 µg mL^−1^) to 108% (**2d** at 300 µg mL^−1^), when compared to the water control. Compound **2a** also promoted significant RG at 75, 50, and 25 µg mL^−1^, with values statistically similar to those of the water control. In contrast, compound **2b** at all concentrations and compound **2e** at 300, 150, 75, and 50 µg mL^−1^ significantly inhibited RG, with values statistically similar to glyphosate and lower than those of the water and DCM controls (Figure 2c).

RG is a sensitive variable that can be influenced either positively or negatively, depending on whether growth is stimulated or inhibited [16, 21]. Deng et al. [22] reported that triazoles can induce hormonal instability by interacting with the isoprenoid metabolic pathway in plants, thereby affecting plant hormone biosynthesis. Consequently, triazoles modulate abscisic acid and gibberellin biosynthesis, thereby inhibiting radicle growth [23].

Regarding compound **2h**, it is worth noting that it inhibited the germinative variable GSI, in contrast to the results reported by Barcelos et al. [21]. Those researchers evaluated glycerol‐derived 1,2,3‐triazoles and observed no change in GSI with the fluorinated derivative. However, the RG response was similar. Barcelos and collaborators also observed reduced aerial development in plants treated with the glycerol‐derived molecule, classifying it as a potential post‐emergent herbicide.

The most phytotoxic derivatives were **2b** and **2e**, which inhibited GSI and also significantly reduced seedling RG (Figure 2c). This result demonstrates the plasticity of these compounds, showing initial pre‐emergent activity.

This sequential effect may be due to the seeds being soaked in the test compound after germination had started, allowing high compound absorption. Similarly, the root is the first plant organ to make direct contact with the compound, as it consumes nutrients and water retained in the seed, making it the most sensitive variable in toxicity assays [24].

The effects observed with compound **2e** may result from a synergistic effect between eugenol and the Cl group position. Chen et al. [25], who studied the phytotoxicity of 1‐allyl‐3‐methylimidazolium chloride, attributed its effects to the presence of the Cl group. They reported that Cl stimulates the hormonal cascade, maintaining seed dormancy by inducing the expression of genes involved in ethylene and abscisic acid biosynthesis and release. This hormonal alteration inhibits germination parameters and may be responsible for the delay in seed germination observed in the present investigation. Another effect of the Cl group is the inactivation of growth‐promoting genes, resulting in reduced radicle length [25].

These macroscopic results may also be associated with and clarified by the activity observed in meristematic cells, as macroscopic parameters are simply the expression of microscopic alterations within the organism resulting from its interaction with the external environment [24].

**FIGURE 3:**
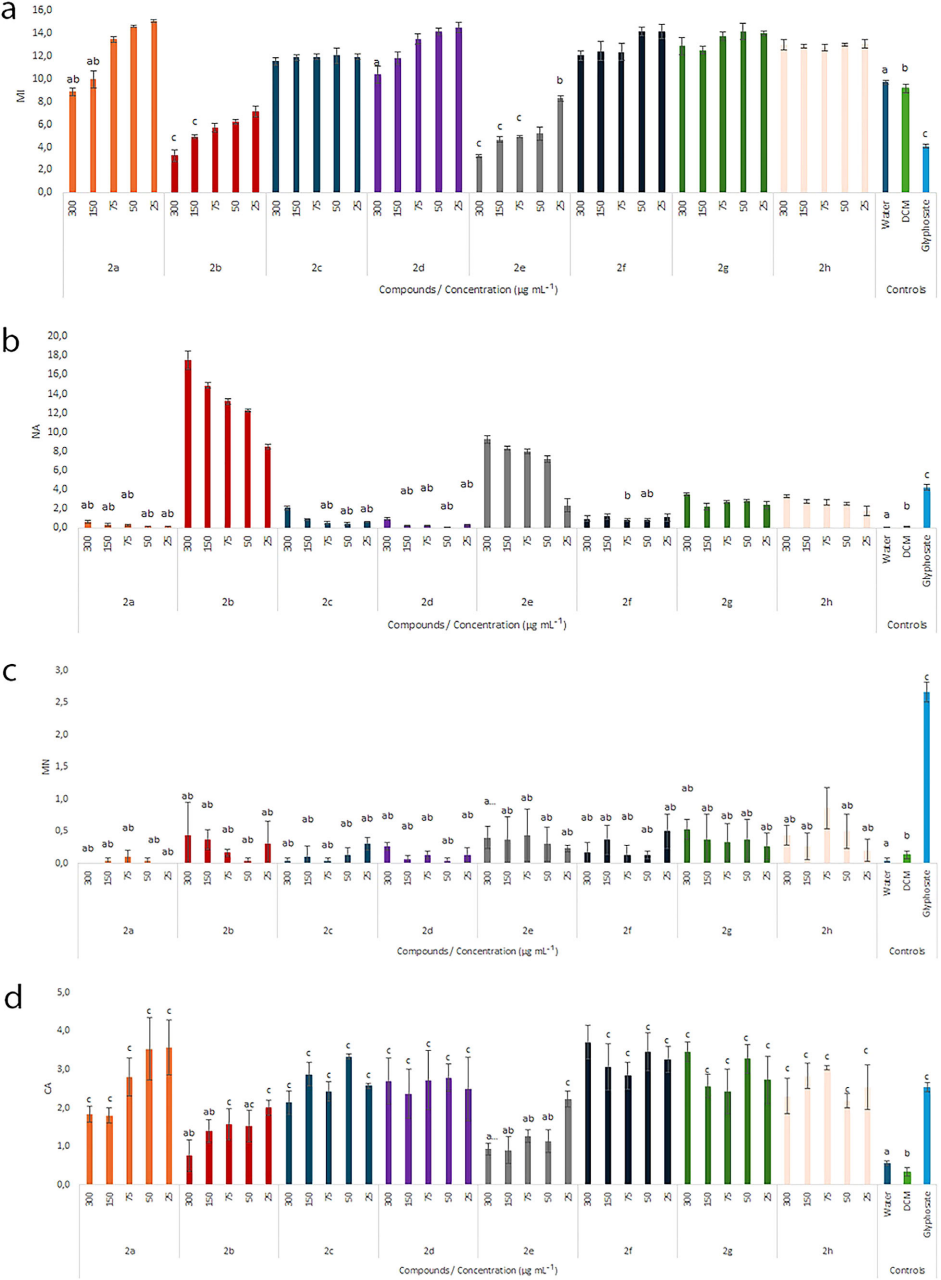
Mitotic index (MI) (a), nuclear alterations (NA) (b), micronuclei (MN) (c), and chromosomal alterations (CA) (d) of meristematic cells obtained from *Lactuca sativa* roots (*n* = 10) treated with triazole compounds **2a–2h** after 48 h of exposure. Means followed by the same letter indicate no significant difference from the respective control groups, and means followed by the absence of letters indicate a difference from all controls, according to Dunnett's test (*p* value < 0.05): (a) negative control (water), (b) negative control (dichloromethane—DCM), (c) positive control (glyphosate).

Figure 3a shows that the eugenol derivatives **2c**, **2f**, **2g**, and **2h** promoted MI induction at all concentrations evaluated, whereas **2a** induced MI at the three lowest concentrations, and **2d** at the four lowest concentrations. MI induction ranged from 19% (**2c** at 300 µg mL^−1^) to 55% (**2a** at 25 µg mL^−1^) compared to the negative control. In contrast, compound **2b** caused a reduction in MI at all concentrations tested, whereas **2e** reduced MI at the four highest concentrations, with inhibition rates ranging from 26% (**2b** at 25 µg mL^−1^) to 67% (**2e** at 300 µg mL^−1^).

The MI reflects the rate of cell proliferation, and both its induction and inhibition are recognized as indicators of environmental toxicity [26]. For MI induction to be considered an indication of the cytotoxicity of the test agent, it must be accompanied by an increase in the frequencies of chromosomal alteration (CA) and NA [16, 20]. This was observed for the compounds that induced MI in the present study (Figure 3a). Therefore, in this case, the MI increase may be related to the organism's attempt to overcome the stress condition [24].

Conversely, MI reduction indicates a lower frequency of cells entering mitosis, which may result either from an increased number of cells remaining in interphase or from an increased number of cells undergoing cell death, as reflected by the NA parameter [16].

According to Luber et al. [26], the MI is directly related to plant development, especially when the response variable is RG. This fact was observed in the two compounds that caused the most significant reduction in growth (**2b** and **2e**). These compounds showed a decrease in both variables, confirming their inhibitory activity in plant development.

Regarding NAs, an increase in NA frequency was observed at all concentrations of **2b**, **2e**, **2f**, **2g**, and **2h**. Increases were also noted in meristematic cells treated with **2c** at 300 and 150 µg mL^−1^ and with **2d** at 300 µg mL^−1^ (Figure 3b). These changes may be associated with the presence of a halogen group, as reported by Barcelos et al. [21], who also observed an increase in NA in studies on fluorinated 1,2,3‐triazoles derived from glycerol. Furthermore, NAs are indicators of the cytotoxicity of the test compound, as they result from biochemical and/or morphological changes occurring in the cell nucleus, associated with mechanisms to correct genetic damage [16].

An increase in micronucleus nucleus (MN) (Figure 3c) was observed with derivative **2h** at 75 µg mL^−1^; however, this increase was significantly lower than that observed for the positive control. This indicates that, for this parameter, the treatments were less toxic than the commercial herbicide. MNs arise from chromosomal fragments or whole chromosomes that failed to be incorporated into the central nucleus during cell division and are subsequently enclosed by a membrane for elimination from the cell [16]. Therefore, they reflect the loss and elimination of genetic material and serve as indicators of the compound's genotoxic action.

All concentrations of compounds **2a**, **2c**, **2d**, **2f**, **2g**, and **2h**, as well as **2b** at the four lowest concentrations and **2e** at 25 µg mL^−1^, led to an increase in the frequency of CAs (Figure 3d). This type of alteration is caused by genotoxic agents that come into contact with the organism and induce changes in the chromosomes, which may be structural—altering the DNA sequence—or numerical—modifying the number of chromosomes passed from the mother cell to the daughter cells [16, 21]. The analysis of the frequency of each CA observed in the treatments allows the identification of the cellular mechanism of action of the test agent, which may be aneugenic and/or clastogenic.

Another NA observed in the meristematic cells was the condensed nucleus (CN), which increased at all concentrations of **2b**, **2e**, **2f**, **2g**, and **2h**; at 300 µg mL^−1^ for **2a** and **2d**; and at 300, 150, and 75 µg mL^−1^ for **2c** (Table 1). CNs differ from interphase cells (G1, S, G2 phases) by presenting a smaller nuclear diameter and greater nuclear staining intensity. This alteration serves as cytological evidence of ongoing cell death [6].

**TABLE 1:** Nuclear (condensed nucleus—CN) and chromosomal alterations (multipolar, adherent, c‐metaphase, bridges, and fragments) of meristematic cells obtained from *Lactuca sativa* roots (*n* = 10) treated with triazole compounds **2a–2h** after 48 h of exposure.

Compound	CN		Multipolar		Adherent		C‐metaphase		Bridges		Fragment	
**2a**	0.60 ± 0.1		0.00 ± 0.00	abc	7.13 ± 1.54	ab	2.65 ± 0.76	abc	2.24 ± 1.07	ab	1.57 ± 2.72	ab
	0.23 ± 0.15	ab	0.37 ± 0.63	abc	6.78 ± 3.56	ab	2.71 ± 0.72	abc	0.96 ± 0.95	ab	1.65 ± 1.05	ab
	0.20 ± 0.10	ab	0.99 ± 0.44	abc	7.65 ± 4.06	ab	0.73 ± 0.72	abc	2.48 ± 0.46	ab	1.24 ± 1.14	ab
	0.07 ± 0.06	ab	0.91 ± 0.40	abc	8.45 ± 1.46	a	2.52 ± 1.44	abc	2.51 ± 0.79	ab	0.45 ± 0.39	ab
	0.10 ± 0.01	ab	0.67 ± 1.15	abc	9.06 ± 1.96	a	0.89 ± 1.54	abc	5.08 ± 1.64	ab	0.00 ± 0.00	ab
**2b**	17.12 ± 0.46		0.00 ± 0.00	abc	8.14 ± 1.13	ab	2.94 ± 2.69	abc	1.75 ± 3.04	ab	0.00 ± 0.00	ab
	14.53 ± 0.35		1.39 ± 2.40		9.56 ± 1.48		4.70 ± 2.92	c	1.35 ± 1.17	ab	3.39 ± 1.14	abc
	13.06 ± 0.33		0.00 ± 0.00	abc	10.13 ± 4.41		4.65 ± 0.76	c	1.83 ± 1.89	ab	3.41 ± 4.37	abc
	12.26 ± 0.14		0.00 ± 0.00	abc	10.12 ± 2.15		2.67 ± 2.41	abc	0.52 ± 0.90	ab	1.06 ± 1.83	ab
	8.20 ± 0.10		0.00 ± 0.00	abc	11.53 ± 3.76	c	4.30 ± 2.60	c	1.93 ± 2.21	ab	0.48 ± 0.84	ab
**2c**	2.03 ± 0.21		0.29 ± 0.51	abc	6.68 ± 2.76	ab	1.45 ± 1.83	abc	2.85 ± 1.89	ab	0.88 ± 0.88	ab
	0.73 ± 0.06		0.28 ± 0.48	abc	8.16 ± 3.52	ab	0.55 ± 0.48	abc	4.79 ± 1.36	ab	1.13 ± 0.51	ab
	0.47 ± 0.21		0.28 ± 0.49	abc	6.99 ± 1.32	ab	1.11 ± 0.96	abc	2.80 ± 1.30	ab	1.38 ± 1.70	ab
	0.20 ± 0.17	ab	0.00 ± 0.00	abc	7.51 ± 1.12	ab	0.57 ± 0.50	abc	6.12 ± 0.80	a	1.65 ± 0.82	ab
	0.33 ± 0.06	ab	0.00 ± 0.00	abc	7.84 ± 1.18	ab	1.69 ± 1.46	abc	2.23 ± 0.44	ab	1.71 ± 2.29	ab
**2d**	0.63 ± 0.12		0.00 ± 0.00	abc	7.04 ± 1.76	ab	1.30 ± 0.62	abc	4.39 ± 2.59	ab	4.20 ± 2.55	c
	0.17 ± 0.06	ab	0.28 ± 0.48	abc	6.77 ± 2.11	ab	2.25 ± 0.40	abc	1.97 ± 2.07	ab	2.25 ± 1.70	abc
	0.03 ± 0.06	ab	0.25 ± 0.43	abc	6.18 ± 1.71	ab	0.99 ± 1.15	abc	3.70 ± 0.84	ab	0.75 ± 0.75	ab
	0.00 ± 0.00	ab	0.00 ± 0.00	abc	8.68 ± 2.64	a	1.43 ± 1.91	abc	3.48 ± 2.74	ab	0.69 ± 1.19	ab
	0.17 ± 0.06	ab	0.00 ± 0.00	abc	6.85 ± 2.37	ab	0.45 ± 0.39	abc	2.49 ± 1.69	ab	0.70 ± 0.71	ab
**2e**	8.84 ± 0.20		1.01 ± 1.75	abc	7.13 ± 7.61	ab	12.57 ± 3.52		3.23 ± 5.59	ab	0.00 ± 0.00	ab
	7.69 ± 0.15		0.00 ± 0.00	abc	4.32 ± 2.23	ab	3.64 ± 2.62	abc	5.12 ± 3.47	ab	0.00 ± 0.00	ab
	7.44 ± 0.24		0.00 ± 0.00	abc	7.48 ± 2.36	ab	2.72 ± 1.18	abc	3.42 ± 1.23	ab	0.69 ± 1.20	ab
	6.77 ± 0.42		0.00 ± 0.00	abc	10.31 ± 2.73		0.72 ± 1.26	abc	1.87 ± 1.92	ab	0.64 ± 1.11	ab
	2.10 ± 0.70		1.19 ± 1.19		9.72 ± 2.68		0.81 ± 0.70	abc	4.42 ± 3.62	ab	0.40 ± 0.69	ab
**2f**	0.77 ± 0.15		0.28 ± 0.48	abc	9.11 ± 1.36	a	1.37 ± 0.44	abc	6.33 ± 1.89	a	1.38 ± 0.95	ab
	0.83 ± 0.16		0.00 ± 0.00	abc	6.97 ± 0.20	ab	1.35 ± 0.48	abc	5.14 ± 1.19	ab	1.34 ± 0.92	ab
	0.69 ± 0.01		0.27 ± 0.46	abc	6.80 ± 3.08	ab	0.52 ± 0.45	abc	4.52 ± 0.66	ab	0.79 ± 0.75	ab
	0.63 ± 0.11		0.72 ± 0.73	abc	7.05 ± 0.97	ab	1.93 ± 2.24	abc	5.11 ± 2.06	ab	1.21 ± 1.52	ab
	0.57 ± 0.21		0.22 ± 0.38	abc	10.10 ± 4.09		0.24 ± 0.42	abc	2.78 ± 1.27	ab	0.95 ± 0.43	ab
**2g**	2.92 ± 0.14		0.25 ± 0.42	abc	10.44 ± 2.60	c	2.07 ± 0.88	abc	4.11 ± 0.70	ab	1.29 ± 0.88	ab
	1.80 ± 0.20	c	0.00 ± 0.00	abc	11.44 ± 3.08	c	0.53 ± 0.46	abc	2.62 ± 1.92	ab	1.58 ± 1.60	ab
	2.27 ± 0.15		0.24 ± 0.41	abc	6.33 ± 1.25	ab	0.25 ± 0.43	abc	3.86 ± 1.57	ab	2.19 ± 1.95	abc
	2.30 ± 0.10		0.00 ± 0.00	abc	7.62 ± 3.49	ab	1.17 ± 2.03	abc	4.29 ± 1.46	ab	1.85 ± 1.37	ab
	2.10 ± 0.17		0.00 ± 0.00	abc	5.50 ± 1.54	ab	1.20 ± 1.10	abc	2.37 ± 1.46	ab	1.67 ± 1.10	ab
**2h**	2.90 ± 0.20		0.00 ± 0.00	abc	6.44 ± 1.40	ab	1.27 ± 0.86	abc	3.82 ± 1.45	ab	0.51 ± 0.87	ab
	2.46 ± 0.20		0.78 ± 0.01	abc	9.81 ± 1.88		0.78 ± 0.78	abc	2.32 ± 0.76	ab	0.78 ± 0.78	ab
	1.79 ± 0.18	c	0.00 ± 0.00	abc	7.04 ± 2.25	ab	1.55 ± 0.75	abc	3.62 ± 1.12	ab	1.06 ± 1.22	ab
	1.99 ± 0.17		0.00 ± 0.00	abc	4.09 ± 1.60	ab	0.77 ± 0.77	abc	1.79 ± 1.16	ab	1.02 ± 0.44	ab
	1.57 ± 0.45	c	0.00 ± 0.00	abc	8.35 ± 3.75	ab	1.55 ± 2.05	abc	2.02 ± 1.12	ab	1.03 ± 1.20	ab
Water	0.00 ± 0.00	a	0.00 ± 0.00	a	1.72 ± 0.61	a	0.00 ± 0.00	a	1.37 ± 0.58	a	0.00 ± 0.00	a
DCM	0.00 ± 0.00	b	0.00 ± 0.00	b	0.73 ± 0.64	b	0.36 ± 0.63	b	0.71 ± 0.62	b	0.00 ± 0.00	b
Glyphosate	1.57 ± 0.15	c	0.00 ± 0.00	c	18.02 ± 5.54	c	1.61 ± 1.39	c	17.31 ± 4.71	c	5.84 ± 4.02	c

*Note*: Means followed by the same letter indicate no significant difference from the respective control groups, and means followed by the absence of letters indicate a difference from all controls, according to Dunnett's test (*p* < 0.05): (a) negative control (water); (b) negative control (dichloromethane—DCM); (c) positive control (glyphosate).

An increase in multipolarity was observed in the treatments with **2b** at 150 µg mL^−1^ and **2e** at 25 µg mL^−1^. Meanwhile, the frequency of c‐metaphase increased in treatments with **2b** at 150, 75, and 25 µg mL^−1^ and with **2e** at 300 µg mL^−1^. Adherence (Table 1) showed a significant increase in treatments with **2b** at 150, 75, 50, and 25 µg mL^−1^, **2e** at 50 and 25 µg mL^−1^, **2f** at 25 µg mL^−1^, and **2h** at 150 µg mL^−1^ (Table 1). This is the most frequent alteration observed in cytogenotoxicity assessments of 1,2,3‐triazoles, demonstrating a pattern in the action of this class of compounds [19, 20, 21].

The frequencies of laggard and bridge CA did not change significantly across any of the treatments evaluated. However, the frequency of fragments (Table 1) showed a substantial increase in **2d** at 300 µg mL^−1^ (Table 1). These are alterations characteristic of clastogenic agents, which act directly on the individual's DNA sequence. In this type of change, the interaction of the test agent with DNA results in the loss of part of the chromosome's DNA. This mechanism of action can be observed with chromosomal fragments and chromosomal bridges [16, 21].

Knowledge of the physicochemical properties of bioactive compounds, such as water solubility and the octanol‐water partition coefficient (log *P*), is a critical determinant of a compound's efficacy, environmental behavior, and safety profile [27]. Water solubility influences how an agrochemical dissolves, transports, and interacts within biological systems and the environment. Highly soluble compounds are more readily taken up by plants and pests, thereby enhancing their effectiveness. However, excessive solubility can lead to rapid leaching into groundwater, posing environmental risks. Conversely, compounds with low solubility may persist in the environment, potentially leading to bioaccumulation. Therefore, achieving an optimal balance in solubility is essential for both efficacy and environmental safety [27].

The log *P* value indicates a compound's lipophilicity, reflecting its ability to traverse lipid membranes. Compounds with moderate log *P* values (typically between 1 and 3) are generally more effective, as they can penetrate biological membranes without accumulating excessively in fatty tissues. Extremely high log *P* values may lead to bioaccumulation and toxicity, whereas very low values can result in poor membrane permeability and reduced efficacy [28].

Considering this aspect, solubility and log *P* theoretical calculations were performed for compounds **2a–2h**, and the results are shown in Table 2.

**TABLE 2:** Theoretical calculations of water solubility and log *P* for compounds **2a–2h**.

Compounds	Δ*G*°_(water)_ (cal mol^−1^)	Δ*G*°_(octanol)_ (cal mol^−1^)	log *P*	Classification considering the solubility
**2a**	−32 980.50	−34 982.93	1.47	Lipophilic
**2b**	−34 058.31	−33 953.59	−0.08	Hydrophilic
**2c**	−33 609.27	−35 353.99	1.28	Lipophilic
**2d**	−34 540.94	−34 754.46	0.16	Lipophilic
**2e**	−34 786.78	−33 780.98	−0.74	Hydrophilic
**2f**	−32 756.98	−34 426.10	1.22	Lipophilic
**2g**	−32 965.02	−32 808.23	−0.11	Hydrophilic
**2h**	−32 052.82	−33 618.07	1.15	Lipophilic

The ab initio calculations revealed that compounds **2b**, **2e**, and **2g** exhibit hydrophilic behavior, whereas molecules **2a**, **2c**, **2d**, **2f**, and **2h** are lipophilic. This indicates that these compounds are likely to exhibit distinct biochemical behaviors within cellular environments. Regarding the chemical structures of the hydrophilic compounds, halogen atoms are present in the structures of **2b** and **2e**, whereas **2g** does not contain any halogen in its structure. The other compounds, in contrast, possess halogens at different positions on the aromatic ring attached to the triazole moiety.

According to Ogawa et al. [29], halogens such as fluorine, chlorine, and bromine have been widely employed in agrochemical synthesis, with their use increasing significantly since the 20th century. In addition to halogen substituents, heterocyclic compounds—especially triazole derivatives—have shown considerable agronomic relevance, displaying a range of biological activities, including bactericidal, fungicidal, and herbicidal effects [19, 20].

Regarding theoretical solubility, compounds **2b** and **2e** were identified as hydrophilic, meaning that their ability to interact with polar environments inside plant cells likely contributed to GSI inhibition and RG reduction in the seedlings. Both **2b** and **2e** were modified with halogen atoms—bromine in **2b** and chlorine in **2e**. Considering that the atomic radius of chlorine is smaller than that of bromine, it is plausible that steric effects may have influenced the degree of hydrophilicity between the compounds; **2b** showed a calculated partition coefficient (log *P*) of −0.08, whereas **2e** had a value of −0.74—a difference of 89% relative to **2e**.

Among halogens commonly used in agrochemicals, fluorine is particularly noteworthy due to its similarity in size to hydrogen and its high electronegativity, which allows it to form stable covalent bonds. This characteristic may help explain the increased interaction potential observed in compounds containing bromine and chlorine [29, 30].

Another relevant observation from the computational data was the correlation between molecular polarity and changes in the frequency of NAs in plant cells. The most significant effects were observed for compounds **2b** and **2e**—hydrophilic compounds that relatively increased NA values in seedlings. In contrast, the lipophilic derivatives **2a**, **2c**, **2d**, and **2f** caused reductions in these values. In this context, it is plausible that **2b** and **2e**, due to their chemical structure, induced mutations in plant cells, leading to changes in NA. This is consistent with the known ability of bromine and chlorine to generate free radicals and, consequently, reactive bromine and chlorine species.

In fields such as chemistry, biology, agriculture, and environmental sciences, PCA is particularly valuable for analyzing multivariate data, allowing researchers to uncover relationships between samples and variables, reduce noise, and facilitate the interpretation of large experimental datasets. Its ability to condense data while preserving essential variance makes PCA a key technique in exploratory data analysis and decision‐making. The PCA, performed on the solubility, phytotoxicity, and cytogenetic data for compounds **2a–2h**, revealed clear groupings.

**FIGURE 4:**
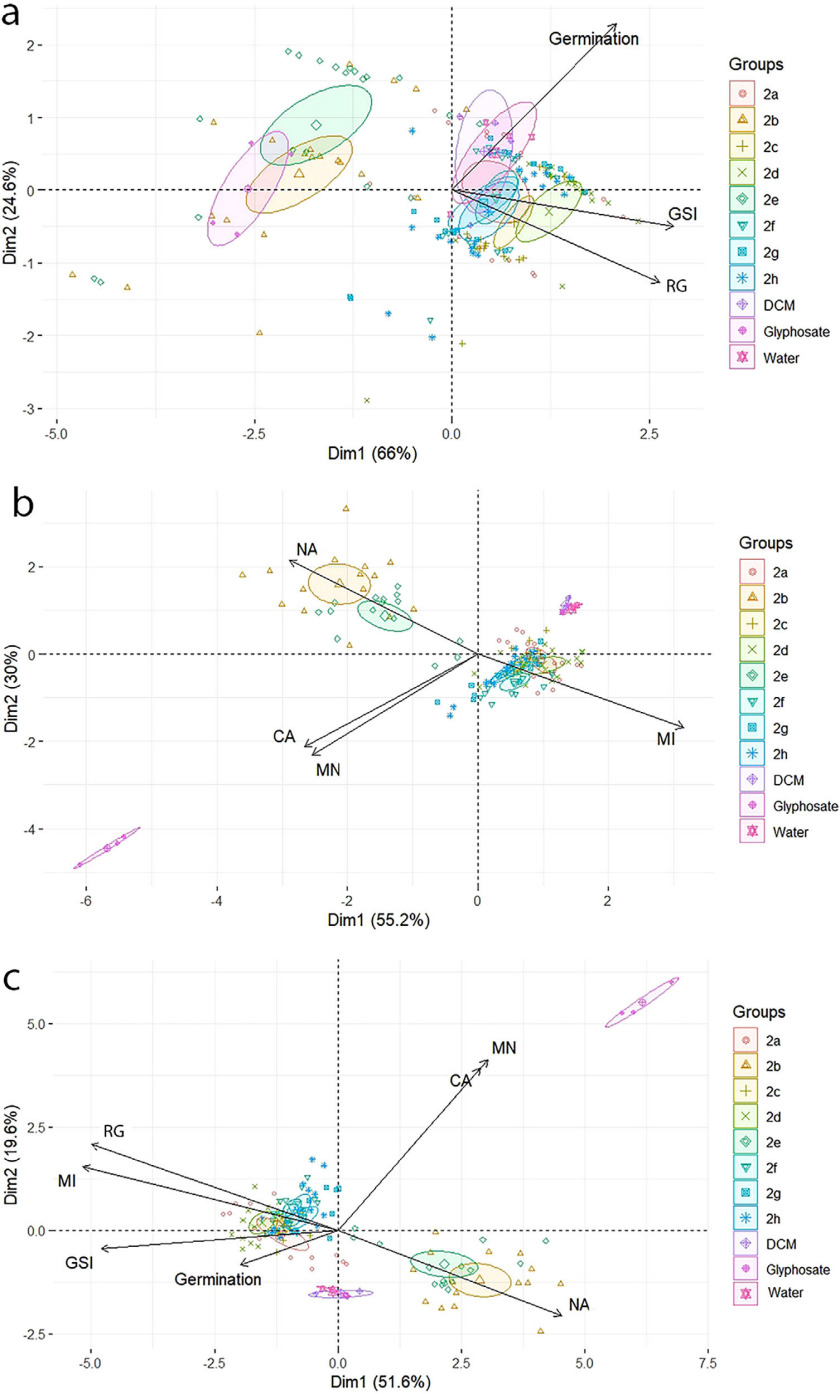
Scatter plot of phytotoxic properties (germination percentage, germination speed index [GSI], and root growth [RG]) (a), cytotoxic properties (nuclear alterations [NA], chromosomal alterations [CA], and micronuclei [MN]) (b), and the correlation between phytotoxicity and cytotoxicity parameters (c) obtained by a function of components 1 (Dim1) and 2 (Dim2).

In the phytotoxicity PCA (Figure 4a), three distinct clusters were identified. A cluster of lipophilic compounds was observed, which promoted RG and GSI, differentiating them from the negative controls, which primarily influenced germination. For the hydrophilic substances (**2b** and **2e**), a cluster was observed near the positive control, suggesting a similar mode of action to glyphosate.

In the cytotoxicity PCA, three clusters also emerged (Figure 4b). The hydrophilic derivatives were associated with NAs, whereas the positive control showed a higher incidence of micronuclei and chromosomal abnormalities.

By correlating the PCA analyses of phytotoxicity and cytotoxicity parameters (Figure 4c), lipophilic compounds were found to promote RG and increase the MI, indicating enhanced seedling development. These effects differed from those observed in the negative controls, which mainly affected germination (Figure 4c), suggesting the potential of these compounds as plant growth promoters.

Lipophilic compound interactions with weeds were previously studied by Ramos et al. [31], who reported limited interactions and growth inhibition rates of approximately 20%. The authors attributed this reduced effect to the low water solubility of the extracts used, which likely limited their bioavailability.

**FIGURE 5:**
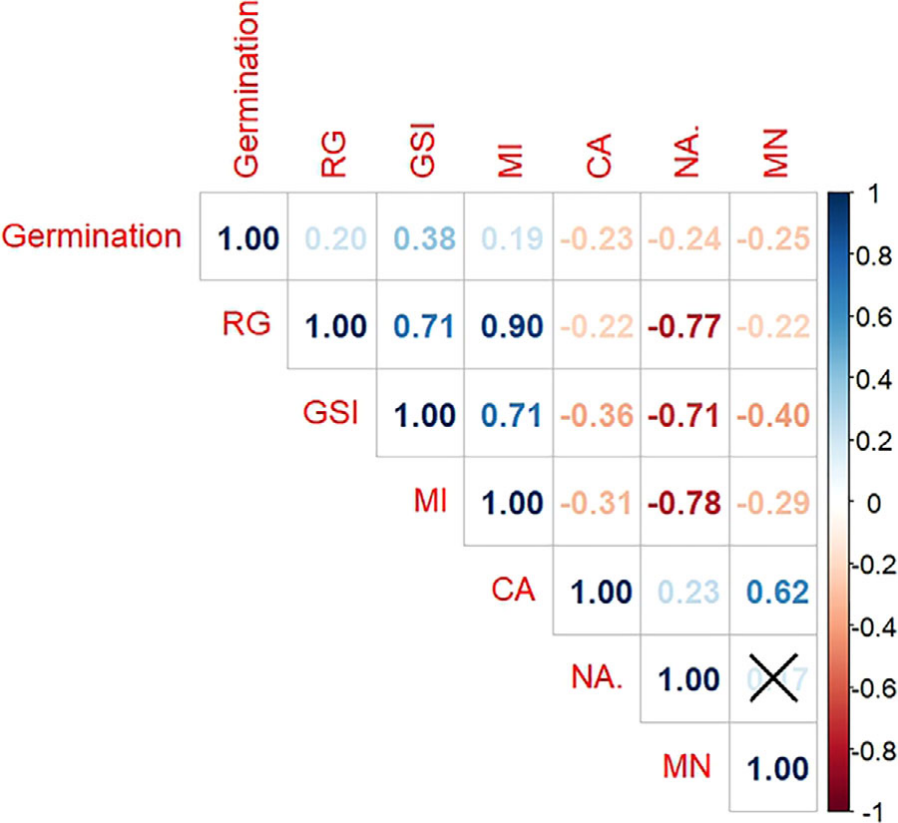
Pearson correlation matrix between phytotoxic and cytogenotoxic variables. Positive correlations (in blue) indicate a directly proportional relationship between variables, whereas negative correlations (in red) indicate inverse relationships. Correlation values range from −1 (perfect negative correlation) to +1 (perfect positive correlation). Black “X” symbols indicate non‐significant correlations based on the *t*‐test for Pearson's correlation (*p* value > 0.05). AN, nuclear alterations; CA, chromosomal alteration; GSI, germination speed index; MI, mitotic index; MN, micronuclei; RG, root growth.

To verify the relationship between MI and RG, a correlation analysis was performed between the phytotoxic and cytotoxic variables, revealing a strong positive correlation (*r* = 0.90), thereby confirming that these variables are directly related. This result supports the conclusion that lipophilic compounds promoted plant development (Figure 5).

For positive correlations, RG and MI showed a strong 0.90 correlation, indicating a very close relationship. This can be explained by the strong association between growth and MI, a result also observed by other authors [2, 8, 25].

**FIGURE 6:**
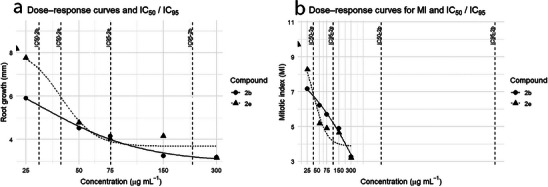
Dose–response curves for root growth (RG) and mitotic index (MI) in roots treated with triazoles **2b** and **2e**. (a) Dose–response curves for RG and corresponding IC_50_ and IC_95_ values. (b) Dose–response curves for MI and corresponding IC_50_ and IC_95_ values. Symbols represent the mean values observed for each concentration, whereas solid and dashed lines correspond to the fitted four‐parameter log–logistic models for compounds **2b** and **2e**, respectively. Vertical dashed lines labeled “IC50‐**2b**,” “IC95‐**2b**,” “IC50‐**2e**,” and “IC95‐**2e**” indicate the estimated concentrations that inhibit RG or MI by 50% and 95%. For MI, the IC_50_ and IC_95_ values estimated for **2b** lie far beyond the tested concentration range, indicating a weaker inhibitory effect on cell proliferation than **2e**.

Given the promising herbicidal potential of the hydrophilic derivatives, their inhibitory effects relative to the negative control were quantified by calculating IC_50_ and IC_95_ values for RG and MI. These parameters represent the concentration required to inhibit plant development by 50% and 95%, respectively (Figure 6).

For triazole **2b**, the IC_50_ for RG was 29.67 µg mL^−1^, whereas MI inhibition required a much higher concentration (IC_50_ ≈ 1676.6 µg mL^−1^). This indicates that **2b** is more strongly associated with phytotoxic effects on root elongation than with cytotoxic effects on meristematic cell proliferation. In agreement with this, the IC_95_ for RG (219 µg mL^−1^) falls within the concentration range tested in this study, whereas the IC_95_ estimated for MI (1.05 × 10^6^ µg mL^−1^) is clearly outside the biologically relevant range, indicating that complete inhibition of the MI was not achieved under the experimental conditions.

For triazole **2e**, a closer relationship between cytotoxicity and phytotoxicity was observed. The IC_50_ values for RG and MI were 39.48 and 35.49 µg mL^−1^, respectively, and the IC_95_ values were 75.53 µg mL^−1^ for RG and 107.93 µg mL^−1^ for MI. Thus, compound **2e** inhibited both endpoints at relatively similar concentrations.

When RG was considered, both **2b** and **2e** showed IC_50_ values close to the lowest concentrations tested; however, **2e** exhibited a lower IC_95_, indicating a stronger inhibitory capacity at higher levels of inhibition. This enhanced effect was also evident for the MI, confirming that **2e** displays greater overall herbicidal potential than **2b**.

The higher biological activity of **2e** may be related to the smaller atomic radius of chlorine, compared with bromine in **2b**. According to Ogawa et al. [29], elements with smaller nuclear radii tend to show stronger interactions with plant cells, and halogens with small radii are widely used in the design of agrochemicals. Elucidating the biological activity and mechanisms of action of new molecules is essential to ensure environmental and human health safety and to support the development of novel agrochemicals that contribute to global food security.

The investigation demonstrated that the hydrophilic compounds bearing halogens (**2b** and **2e**) exhibited the highest herbicidal potential, with **2e** standing out in particular. Additionally, these derivatives showed cellular mechanisms of action characterized as clastogenic and aneugenic. However, further studies are needed to verify the herbicidal activity of these molecules under field conditions.

Molecular docking reveals distinct interaction profiles for triazole–eugenol derivatives. The compounds **2b** and **2e** exhibited physicochemical behavior in the molecular docking assays that differed substantially from that of glyphosate. Unlike glyphosate, which is highly pH‐dependent due to its multiple ionizable groups, **2b** and **2e** do not undergo acid–base equilibrium or ionization, making them pH‐independent. They are also less hydrophilic (log *P*(2b) = −0.08; log *P*(2e) = −0.74) than glyphosate (log *P* = −2.80), which contains highly polar phosphonate, amino, and carboxylic acid groups.

**FIGURE 7:**
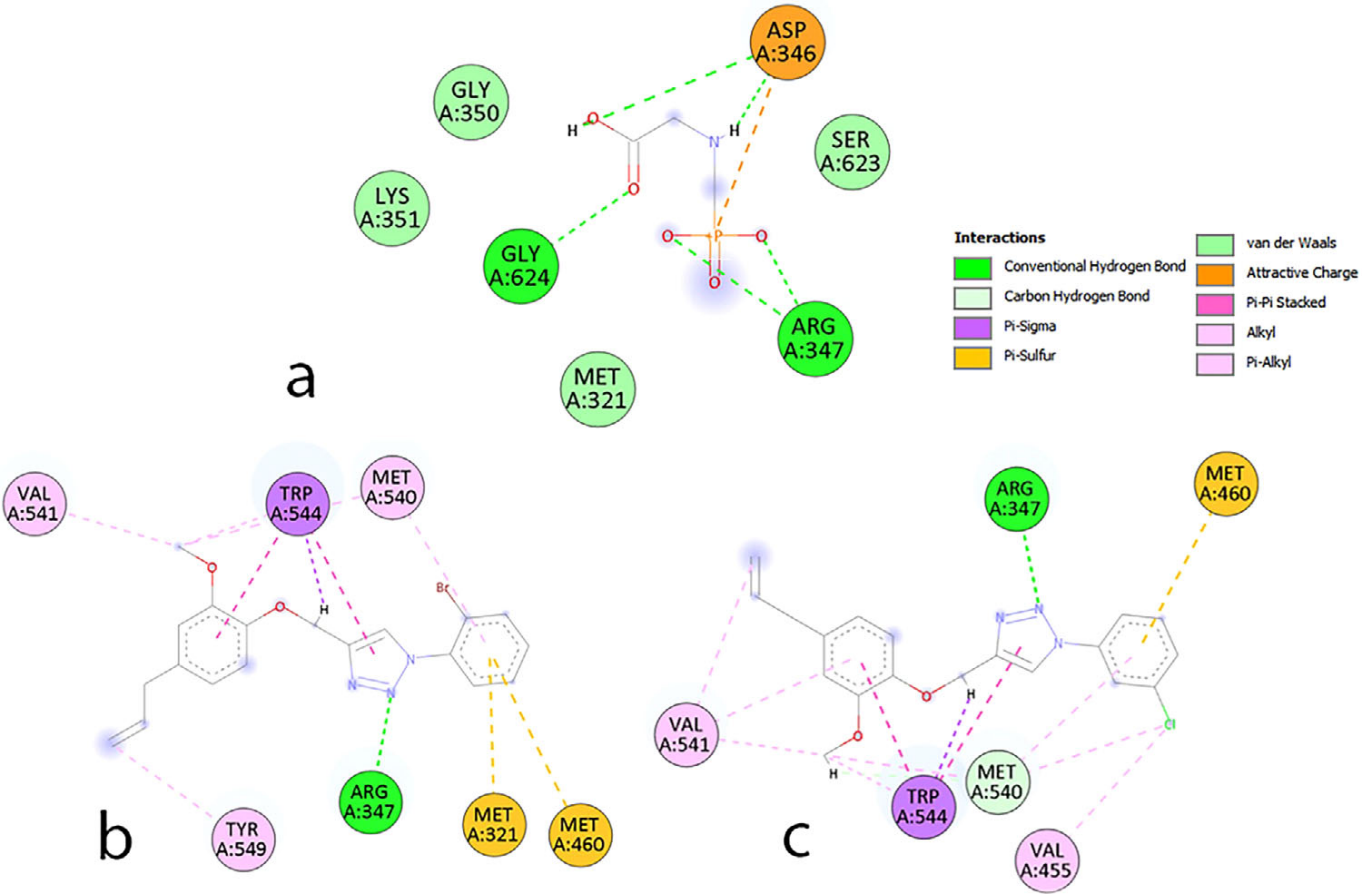
Molecular interactions shown for glyphosate (a), **2b** (b), and **2e** (c).

These physicochemical distinctions directly influence the interaction patterns observed in the LmALS enzymatic cavity. The chemical structures and intermolecular interactions of the LmALS residues are shown in Figure 7.

Glyphosate predominantly forms polar interactions, including electrostatic contacts with ASP346 and conventional hydrogen bonds with GLY624 and ARG347, consistent with its hydrophilic nature. In contrast, **2b** and **2e** display a broader and more diverse set of interactions—not only retaining the common ARG347 contact but also establishing numerous apolar interactions within the enzyme's cavity. This behavior arises from the hydrophobic substituents present on the eugenol‐derived aromatic rings, specifically the *ortho*‐bromine in **2b** and *meta*‐chlorine in **2e**.

Docking calculations indicated that the interaction energies of **2b** and **2e** with LmALS were more negative than that of glyphosate. Although glyphosate exhibits an interaction energy of −3.50 kcal mol^−1^, the triazole‐modified compounds showed values of −5.16 for **2b** and −5.42 kcal mol^−1^ for **2e**, reflecting the enhanced complementarity provided by their structural features. Both molecules form an extensive network of stabilizing interactions—including hydrogen bonds, π–π stacking, π‐alkyl, π‐sulfur, induced dipole interactions, and others—which collectively strengthen binding and contribute to their inhibitory potential toward LmALS.

Regarding the residues involved, ARG347 stands out as the primary polar contributor, whereas apolar residues such as MET460, MET540, TRP544, and VAL541 play key roles in establishing hydrophobic contacts, as detailed in Tables S1–S3. For **2b**, ARG347 interacts with the triazole nitrogens; MET460 associates with the ortho‐brominated aromatic ring; MET540 contacts both the brominated sp^2^ carbon and the methoxy group from the eugenol moiety; and TRP544 engages in multiple interactions with the methylene bridge and aromatic systems. For **2e**, the meta‐chlorine substitution drives π‐sulfur, π‐alkyl, and induced dipole interactions with MET460 and MET540, whereas TRP544 interacts through induced dipole effects involving the allylic and methoxy groups.

Overall, the predominance of apolar interactions in **2b** and **2e**—markedly distinct from the polar interaction profile of glyphosate—promotes improved molecular accommodation within the LmALS catalytic site. The high number of hydrophobic contacts not only compensates but surpasses the intrinsic strength of isolated polar interactions formed by glyphosate, thereby explaining the more favorable docking energies obtained with AutoDock Vina. Collectively, these results demonstrate the strong potential of the triazole‐modified eugenol derivatives **2b** and **2e** for commercial development, as their moderate apolarity and diverse interaction patterns enable herbicidal formulations requiring fewer harsh additives, aligning with principles of green chemistry.

Structure–activity relationship (SAR) analysis correlates electronic and physicochemical properties with docking affinity. The essential SAR parameters for **2a–2h** molecules are shown in Table 3.

**TABLE 3:** Essential structure–activity relationship (SAR) parameters obtained from semiempirical calculations.

Compound	MW (dalton)	log *P*	Dipole moment (debye)	∆*E*_gap_ (eV)	Interaction energy (kcal/mol)	Mulliken charge	COSMO volume (Å^3^)
**2a**	400.27	1.47	2.85	7.57	−5.11	0.03	428.04
**2b**	400.27	−0.08	5.08	7.59	−5.16	0.28	417.11
**2c**	400.27	1.28	3.47	7.65	−5.36	0.60	428.05
**2d**	355.82	0.16	2.76	7.57	−5.24	0.11	420.56
**2e**	355.82	−0.74	3.42	7.66	−5.42	0.54	416.29
**2f**	355.82	1.22	5.86	7.69	−5.09	0.35	411.38
**2g**	321.38	−0.11	4.53	7.83	−5.31	0.83	397.87
**2h**	339.37	1.15	3.15	7.76	−5.21	0.11	405.62

Abbreviation: MW, molecular weight.

The SAR parameters differ significantly from glyphosate. For instance, this molecule is highly hydrophilic (log *P* = −2.80) and pH–dependent due to ionizable phosphonate, amino, and carboxylate groups. On the contrary, compounds **2a–2h** exhibit moderate lipophilicity (log *P* ranging from −0.74 to 1.47) and do not undergo acid–base equilibrium since there is no acid‐base group in their structures. In particular, **2b** (log *P* = −0.08) and **2e** (log *P* = −0.74) exhibit a balanced lipophilic/hydrophilic profile that appears optimal for interaction with the predominantly hydrophobic pocket of LmALS, as discussed in the docking section. Consequently, the lower hydrophilic behavior of **2b** and **2e** molecules favors an increased number of intermolecular interactions, as seen by interaction energies of those molecules over LmALS.

Docking results revealed that all triazole derivatives show more favorable interaction energies with LmALS than glyphosate (−3.50 kcal mol^−1^). Notably, **2e** (Cl–*meta*) displayed the lowest docking energy (−5.42 kcal mol^−1^), followed by **2b** (Br–*orto*, −5.16 kcal mol^−1^) and **2c** (Br–*meta*, −5.36 kcal mol^−1^). In contrast, derivatives with halogens in the *para* position (**2a**, **2d**) exhibited slightly less favorable energies, suggesting that *meta*–substitution improves complementarity with the enzyme's active site.

Calculated quantum–chemical parameters indicate high electronic stability for all compounds (mean ∆*E*_gap_ = 7.67 eV), albeit less stable than glyphosate itself (∆*E*_gap_ = 10.11 eV). The dipole moments ranged from 2.76 to 5.86 D, reflecting charge asymmetry that may facilitate orientation within the active site. Notably, **2e** combined the most significant gap (7.66 eV) with the most favorable docking energy, suggesting that electronic stability and optimal halogen placement (e.g., chlorine) synergistically enhance binding. The same conclusion holds for the **2b** molecule, with bromine in the *ortho* position, albeit with slightly lower interaction energy. Considering the docking energy from AutoDock Vina, Table 4 shows the correlations between this variable and other SAR data.

**TABLE 4:** Calculated Pearson correlation factor of selected structure–activity relationship (SAR) parameters with docking energy.

Parameter	Pearson correlation factor	Conclusion^a^
MW	+0.217	Very weak positive trend
log *P*	+0.549	Moderate‐strong positive trend
Dipole moment	+0.344	Weak positive trend
∆*E*_gap_	−0.154	Very weak positive trend
Mulliken charge	−0.654	Moderate negative trend
COSMO volume	−0.237	Weak negative trend

Abbreviation: MW, molecular weight.

^a^ Correlation strength: |*r*| < 0.3 (weak), 0.3 ≤ |*r*| < 0.7 (moderate), |*r*| ≥ 0.7 (strong). Positive *r* indicates that higher parameter values correlate with less favorable (less negative) docking energies.

In Table 4, the Pearson correlation shows a weak‐to‐moderate trend in the calculated parameters shown in Table 3. Given the small sample size (*n* = 8), none of the correlations reached statistical significance at *α* = 0.05 (all *p* values > 0.05), and they should be interpreted as preliminary trends. In this context, the correlation between physicochemical parameters and docking energies showed that the mean Mulliken charge was the most strongly correlated with the interaction energy of the LmALS. On the other hand, the log *P* showed a moderate, positive trend with docking energy, and other parameters had weak to no correlation, with *r* values below 0.35, suggesting a secondary influence on the binding of the modified molecules during inhibition of the LmALS enzyme. These statistical trends align with the intermolecular interactions observed in docking poses, notably the negative correlation between Mulliken charge and docking energy (*r* = −0.654), being consistent with the prevalence of apolar interactions in the best performance ligands **2b** and **2e**, where less harmful atomic charges appear to favor hydrophobic interactions in residues such as MET460 and TRP544. The moderate negative correlation with Mulliken charge suggests that molecules with more negative (or less positive) partial atomic charges tend to bind more strongly. This is chemically plausible because such charge distributions can enhance polarizability and favor apolar interactions (e.g., π‐alkyl and induced dipole) with hydrophobic residues in the LmALS active site, as seen in the docking poses of **2e** and **2b**. The correlation trends align with the interaction patterns observed in the docking poses. For instance, the meta‐chlorine in **2e**, which contributes to a less negative Mulliken charge, forms π‐sulfur and π‐alkyl interactions with MET460 and MET540, which explain its superior docking score. In contrast, the *para*‐substituted derivatives, despite similar molecular weight (MW) and volume, lack these specific apolar contacts, resulting in weaker binding.

## 3 Conclusions

The results of this study demonstrate that eugenol‐derived compounds containing the 1,2,3‐triazole group show significant potential as bioactive molecules of agronomic interest. Among the eight molecules evaluated, compounds **2b** and **2e** stood out for exhibiting pronounced phytotoxic effects, with significant inhibition of *L. sativa* germination and RG at levels comparable to those of the commercial herbicide glyphosate. These same compounds also reduced the MI and increased CA and NAs, indicating cytotoxic and genotoxic potential. The correlation between physicochemical properties and biological responses suggests that the hydrophilicity of **2b** and **2e** may enhance their interactions with plant tissues, contributing to the observed efficacy. Therefore, these findings reinforce the potential of these derivatives as promising candidates for the development of alternative herbicides that act through mechanisms distinct from conventional synthetic products and provide a basis for future studies on their selectivity, mode of action, and environmental safety.

## 4 Experimental Section

### 4.1 Synthesis of Eugenol‐Derived Triazoles

For completeness, details regarding the preparation and structural characterization data of the compounds herein investigated can be found in Figures S1–S43.

### 4.2 Biological Assays

#### 4.2.1 Plant Material

The eucotyledonous plant *L. sativa* L. “Crespa Grand Rapids” (Isla Pak) was used as the plant model. The seeds were within the expiration date, 100% pure, and had a germination rate of 99.7%. The experiments with the plant model were conducted in the Laboratory of Cytogenetics and Plant Tissue Culture located at the Federal University of Espírito Santo—Alegre Campus, ES, Brazil.

#### 4.2.2 Test Solutions and Controls

The eugenol derivatives **2a–2h** were diluted into five concentrations: 300, 150, 75, 50, and 25 µg mL^−1^. As negative controls, distilled water and the organic solvent DCM—used in the preparation of the test solutions—were employed. The commercial herbicide glyphosate, marketed as “Roundup,” at a concentration of 1 mg mL^−1^, was used as a positive control, given that it is the most widely applied agrochemical of this class worldwide [16].

#### 4.2.3 Initial Macroscopic Analysis of the Parameters of Seed Soaking

For the initial macroscopic analysis of seed soaking, the continuous application method was used, with test solutions applied directly to the seeds to assess their effect on germination. To do this, 25 seeds were placed in Petri dishes (9 cm) lined with filter paper. Then, 2.5 mL of the corresponding treatment solution was added to each dish, and 10 min were allowed for the solution to evaporate. After evaporation, 2.5 mL of distilled water was added to each dish to maintain moisture, and the dishes were sealed with plastic film. The experiment followed a completely randomized design with four replicates. Germination was assessed every 8 h during the first 48 h to evaluate the GSI. After the initial 48 h of exposure, the final GP and RG were assessed using a digital caliper to evaluate the root development of all seedlings.

#### 4.2.4 Cytogenotoxicity Analysis

To analyze the effects of the treatments on mitotic division and chromosomes in the model plant, 10 roots from each treatment group were collected after 48 h of exposure and fixed in Carnoy's solution, with two solution changes: the first after 10 min of storage at −20°C, and the second after 24 h. Slides were prepared using the squash technique and stained with 2% acetic orcein (v v^−1^), with one slide prepared per replicate, totaling four slides per treatment. A total of 1000 meristematic cells per slide were evaluated to observe mitotic phases and any chromosomal abnormalities. The variables assessed were as follows:

–MI: number of cells in mitosis/total number of cells,
–CA: total number of cells with chromosomal abnormalities (C‐metaphase, multipolarity, chromosomal adherence, chromosome bridge, lagging chromosome, chromosomal fragment)/total number of cells,
–NA: total number of cells with NAs (CN and MN)/total number of cells. The frequency of each alteration was calculated as the number of dividing cells with that alteration divided by the total number of dividing cells.

#### 4.2.5 Statistical Analysis

All phytotoxicity and cytogenotoxicity variables were tested for residual normality and variance homogeneity using the Shapiro–Wilk and Bartlett tests, respectively. The results from phytotoxicity and cytogenotoxicity analyses were submitted to analysis of variance (ANOVA), and means were compared using Dunnett's test at a 5% significance level. For multivariate analysis, PCA was performed on the correlation matrix to explore the data structure and identify patterns of variation among the analyzed variables. Additionally, Pearson correlation coefficients were calculated, and their significance was evaluated using Student's *t*‐test for the null hypothesis of zero correlation. Dose–response analyses were conducted to estimate inhibitory concentrations (IC_50_ and IC_95_) for RG and MI. Curves were fitted using a four‐parameter log–logistic model (LL.4), and effective doses were calculated using the delta‐method–based confidence intervals implemented in the ED( ) function of the *drc package*. All statistical analyses were performed using R software (version 4.3.3; R Development Core Team, 2024) within the RStudio development environment (version 2024.12.0; RStudio Team, 2024) [32].

### 4.3 Computational Details

The 3D structures of the molecules were initially drawn using Avogadro 1.2.0, and ab initio calculations were performed with the OpenMOPAC package version 22.0.6, employing the PM7 semiempirical method. Calculations were conducted in both water and octanol environments using the COSMO implicit solvation model on a Windows‐based computer equipped with optimized hardware. The optimized 3D structures of the compounds used in the theoretical calculations are shown in Figure S44.

Thermodynamic properties, including the standard absolute entropy (*S*°) and the standard enthalpy change (Δ*H*°) of the molecules, were also calculated to determine the variation in Gibbs free energy, according to the following equation:

(1) $$\Delta G^{\circ }=\Delta H^{\circ }-T\Delta S$$

To calculate the theoretical solubility of the compounds, the following thermodynamic scheme was considered. Assuming the molecules undergo equilibrium processes, the following equations were used:

(2) $$\mathrm{TE}x_{\left(\mathrm{vacuum}\right)}⇄\mathrm{TE}x_{\left(\mathrm{water}\right)}\;∴\;K_{1}=\frac{\mathrm{TE}x_{\left(\mathrm{vaccum}\right)}}{\mathrm{TE}x_{\left(\mathrm{water}\right)}}$$

(3) $$\mathrm{TE}x_{\left(\mathrm{vacuum}\right)}⇄\mathrm{TE}x_{\left(\mathrm{octanol}\right)}\;∴\;K_{2}=\frac{\mathrm{TE}x_{\left(\mathrm{vaccum}\right)}}{\mathrm{TE}x_{\left(\mathrm{ocatnol}\right)}}$$

where TE*x* represents the molecules studied in this work, and *K*_1_ and *K*_2_ are the respective equilibrium constants for each process. Since water and octanol are commonly used for the calculation of partition coefficients, the following equilibrium was also considered (Equation 4):

(4) $$\mathrm{TE}x_{\left(\mathrm{water}\right)}⇄\mathrm{TE}x_{\left(\mathrm{octanol}\right)}\;∴\;\frac{K_{2}}{K_{1}}=\frac{\mathrm{TE}x_{\left(\mathrm{water}\right)}}{\mathrm{TE}x_{\left(\mathrm{ocatnol}\right)}}$$

Here, $P=\frac{K_{2}}{K_{1}}$, where *P* is the partition coefficient of the compound between water and octanol.

The Gibbs free energy change for this equilibrium is given by the following equation:

(5) $$\Delta G_{r}^{\circ }=\Delta G_{\left(\mathrm{octanol}\right)}^{\circ }-\Delta G_{\left(\mathrm{water}\right)}^{\circ }$$

where $\Delta G_{r\;}^{\circ }$ is the Gibbs free energy change of the equilibrium, and $\Delta G_{(\mathrm{ocatnol})\;}^{\circ }$ and $\Delta G_{\left(\mathrm{water}\right)}^{\circ }$ are the Gibbs free energies of the molecule in octanol and water, respectively.

At equilibrium, Gibbs free energy can also be calculated using the following equation:

(6) $$\Delta G_{r}^{\circ }=-\mathrm{RT}\;\ln P$$

where *R* is the universal gas constant (1.98 cal mol^−1^ K^−1^), and *T* is the absolute temperature (298 K). Combining the equations above and considering that lnP=2.303,logP, Equation (7) is obtained:

(7) $$\log P=-\frac{\left(\Delta G_{\left(\mathrm{octanol}\right)}^{\circ }-\Delta G_{\left(\mathrm{water}\right)}^{\circ }\right)}{1363.67}$$

where logP is the thermodynamically derived partition coefficient of the substance in logarithmic scale. It is important to note that the thermodynamic values obtained from the OpenMOPAC calculations were reported in calories per mole of compound.

### 4.4 Docking Analysis

Molecular docking was conducted using the Rowan Scientific platform [33], which employs AutoDock Vina [34], Vinardo [34], and QuickVina 2 [35] algorithms, as well as the AIMNet2 machine learning algorithm [36]. The enzyme was acetolactate synthase (ALS) that comes from *Lolium multiflorum*, hereby named LmALS. ALS, also called acetohydroxyacid synthase, was selected because it is the first enzyme in the biosynthetic pathway of branched‐chain amino acids, including valine, isoleucine, and leucine [37]. Moreover, it is the target of more than 50 commercial herbicides [37]. The coordinates of the enzymatic center were (−6.17, −20.06, −5.55) with a 15 Å × 15 Å × 20 Å rectangle. The enzyme structure was constructed by homology using GenBank (https://www.ncbi.nlm.nih.gov/protein/) with accession number AG30931.1. Using CHai Discovery (https://www.chaidiscovery.com/), the 3D structure of LmALS was acquired, and the homology percentage was obtained using BLAST [38, 39, 40], which shared around 74.14% of similarity with enzyme PDB 3E9Y [41] as homology model in comparison with LmALS. The final enzymatic model included tiamin diphosphate (ThDP), Mg(II) ion, and FAD as cofactors and used in molecular docking as PDBQT format. The most favorable structure of triazole‐modified eugenol shown was drawn using Avogadro 1.20 [42] and geometrized using the PM7 semiempirical method in the MOPAC package [43]. For benchmarking purposes, monosulfuron and glyphosate molecules were included in the PDB enzyme file.

### 4.5 SAR Parameter Calculation and Statistical Correlation Analysis

Key SAR parameters—including MW, partition coefficient (log *P*), dipole moment, HOMO–LUMO gap (∆*E*_gap), mean Mulliken atomic charge, and COSMO volume—were derived from the optimized PM7 geometries using the MOPAC package. Linear correlations between these molecular descriptors and the docking energies were assessed using Pearson's correlation coefficient. The two‐tailed *p* value was calculated using Student's *t*–distribution with *n*‐2 degrees of freedom. Given the preliminary nature of the series (*n* = 8), correlations were considered significant only for *p* < 0.05, but trends with *p* < 0.10 were also discussed as indicative.

## Author Contributions

**Thayllon de Assis Alves** and **Thammyres de Assis Alves**: contributed to the conduction and carrying out of the experiments and participated in the writing of this document and elaboration of figures. **Poliana Aparecida Rodrigues Gazolla**, **Ângela Maria Almeida Lima**, **Mariana Belizario de Oliveira**, and **William dos Santos Belarmino**: contributed to the synthesis of molecules and elaboration of this work, writing and discussion of the results. **Camila Luiz Sena** and **Othon Souto Campos**: contributed to the docking analysis, SAR parameter calculation, and statistical correlation analysis. **Róbson Ricardo Teixeira**, **Adilson Vidal Costa**, **Elias Terra Werner**, and **Milene Miranda Praça‐Fontes**: contributed to the elaboration of this work, data analysis, creation of graphs, writing, and discussion of the results. For manuscript editing and revision, all authors equally contributed to this work and approved the final manuscript version for submission.

## Conflicts of Interest

The authors declare no conflicts of interest.

## Supporting information




**Supporting File 1**: cbdv70902‐sup‐0001‐SuppMat.docx

## Data Availability

The data that support the findings of this study are available from the corresponding author upon reasonable request.
