# Supplementary material for: Cytogenotoxic Effects and Physicochemical and Molecular Profiles of Eugenol‐Derived Triazoles With Phytotoxic Potential
Source: Chem Biodivers. 2026 Jan 28;23(1):e03621. doi: 10.1002/cbdv.202503621 (PMC12849542; doi:10.1002/cbdv.202503621)
Supplement: Supplementary file 1 — Supporting File 1: cbdv70902‐sup‐0001‐SuppMat.docx [file CBDV-23-e03621-s001.docx]

**SUPPLEMENTARY MATERIAL**

Cytogenotoxic Effects and Physicochemical and Molecular Profiles of Eugenol-Derived Triazoles with Phytotoxic Potential

Thayllon de Assis Alves,^a^ Thammyres de Assis Alves,^a^ Poliana Aparecida Rodrigues Gazolla,^b^ Ângela Maria Almeida Lima,^b^ Mariana Belizario de Oliveira,^b^ William dos Santos Belarmino,^b^ Camila Luiz Sena,^b^ Róbson Ricardo Teixeira,^c^ Elias Terra Werner,^a^ Othon Souto Campos,^c^ Adilson Vidal Costa,*^,b^ and Milene Miranda Praça-Fontes*^,a^

^a^ Departamento de Biologia, Universidade Federal do Espírito Santo, Alto Universitário, s/n, Guararema, 29500-000 Alegre, Espírito Santo State, Brazil ([milenemiranda@yahoo.com.br](mailto:milenemiranda@yahoo.com.br))

^b^ Departamento de Química e Física, Universidade Federal do Espírito Santo, Alto Universitário, s/n, Guararema, 29500-000 Alegre, Espírito Santo State, Brazil ([avcosta@hotmail.com](mailto:avcosta@hotmail.com))

^c^ Departamento de Química, Universidade Federal de Viçosa, Av. P.H. Rolfs, s/n, 36570-900 Viçosa, Minas Gerais State, Brazil.

**SUPPLEMENTARY MATERIAL**

**1. CHEMICAL AND INSTRUMENT**

Reagents and solvents were commercially obtained from Sigma-Aldrich (St. Louis, MO, USA), Êxodo Científica (Sumaré, SP, Brazil), and Química Moderna (Barueri, SP, Brazil). Thin-layer chromatography (TLC) analyses were performed on pre-coated silica gel plates with aluminum backing, using different solvent systems. TLC plates were visualized under ultraviolet light (*λ* = 254 nm) and/or potassium permanganate solution. Column chromatography separations were carried out using silica gel (70–230 mesh, Sigma-Aldrich) as the stationary phase. Infrared (IR) spectra were recorded using the attenuated total reflectance (ATR) technique on a Varian 660 instrument (Varian, Palo Alto, CA, USA) equipped with a GladiATr accessory, in the range of 4000 to 500 cm⁻¹. Hydrogen (^1^H NMR, 300 MHz, 400 MHz, and 600 MHz) and carbon (^13^C NMR, 75 MHz, 100 MHz, and 150 MHz) nuclear magnetic resonance spectra were obtained using three spectrometers: VARIAN MERCURY 300 (Varian, Palo Alto, CA, USA), BRUKER AVANCE 400 (Bruker, Billerica, MA, USA), and Premium COMPACT (Bruker, Billerica, MA, USA). The deuterated solvents used were chloroform (CDCl_3_) and dimethyl sulfoxide (DMSO-*d_6_*). NMR data are reported as follows: chemical shift (*δ*) in ppm, multiplicity, number of hydrogens, and scalar coupling constants (*J*) expressed in Hertz (Hz). Multiplicities are abbreviated as follows: s (singlet), d (doublet), dd (double doublet), t (triplet), td (triplet of doublets), ddtap (apparent doublet of doublet of triplet), and m (multiplet). Melting points were determined using an MQAPF-302 instrument (Micro Química, Cotia, Brazil) and were not corrected. Chromatograms for samples **2a**–**2h** were obtained using a Vanquish Flex ultra-high-performance liquid chromatograph (Thermo Scientific, Bremen, Germany) coupled to an LTQ-XL mass spectrometer (Thermo Scientific, Bremen, Germany). A 100 Å C18 column, 150 × 2.1 mm, Luna Omega 1.6 μm (Phenomenex, São Paulo, Brazil) was employed for chromatographic separation, with 2 *μ*L of sample injected at a flow rate of 350 *μ*L min⁻¹. A gradient of 5–95% over 7 min was applied at 60 °C, using water and methanol, both containing 0.1% formic acid. Mass spectra were acquired in the *m/z* range of 100–1500 in positive ionization mode. ESI ion source parameters were as follows: heater temperature (°C): 350; sheath gas flow rate (arb): 30; auxiliary gas flow rate (arb): 10; spray voltage (kV): 4.0; capillary voltage (V): 44.00. LTQ-XL calibration was performed using a CalMix LTQ solution in positive mode, over an m/z range of 100–2000, with an ion accumulation time of 0.005 s and a capillary voltage of 4.0 kV. Mass spectra were processed using the Xcalibur software, version 2.2 (Thermo Scientific, Bremen, Germany). Finally, MS/MS experiments were conducted using 20% normalized collision energy.

**2. SYNTHETIC PROCEDURES AND STRUCTURAL CHARACTERIZATION DATA OF COMPOUND 1 AND TRIAZOLES 2a-2h**

*Synthesis of* *4-allyl-2-methoxy-1-(prop-2-yn-1-yloxy)benzene* (**1**)

A 50 mL round-bottom flask was charged with sodium hydroxide (0.313 g; 7.38 mmol), eugenol (1.20 g; 7.32 mmol), and 25.0 mL of methanol. The resulting mixture was stirred and heated at 40 °C for 30 minutes. After this period, methanol was removed under reduced pressure, and 10.0 mL of absolute ethanol was added to eliminate residual water. The ethanol was then evaporated, and 25.0 mL of acetonitrile was added to the flask under a nitrogen atmosphere, followed by the slow addition of propargyl bromide (800 *μ*L; 8.79 mmol). The reaction mixture was stirred at room temperature for 18 hours. After this time, the reaction volume was reduced, and 25.0 mL of an aqueous sodium hydroxide solution (0.1 mol L⁻¹) was added. The mixture was then transferred to a separatory funnel, and the aqueous phase was extracted with dichloromethane (3 × 25.0 mL). The organic layers were combined, washed with a saturated aqueous solution of sodium chloride (25.0 mL), dried over anhydrous sodium sulfate, filtered, and concentrated under reduced pressure. Compound **1** was purified by silica gel column chromatography eluted with a 4:1 (v v^-1^) hexane–ethyl acetate mixture, yielding a yellow oil with 81% yield (1.20 g; 7.30 mmol). The structure of **1** was secured by the following data. TLC: R_f_ = 0.65 (hexane/ethyl acetate 4:1 v v^-1^); IR (ATR) *v*_max_/cm^-1^ 3291, 3076, 3002, 2935, 2905, 2834, 1638, 1594, 1507, 1452, 1419, 1374, 1334, 1257, 1214, 1138, 1023, 995, 914, 851, 802, 750; ^1^H NMR (300 MHz, CDCl_3_) *δ* 2.49 (t, 1H, *J =* 2.4 Hz), 3.35 (d, 2H, *J =* 6.7 Hz), 3.86 (s, 3H), 4.73 (d, 2H, *J =* 2.4 Hz), 5.06-5.12 (m, 2H), 5.96 (ddt, 1H, *J =* 16.8, 10.2, 6.7 Hz), 6.72─6.74 (m, 2H), 7.00 (d, 1H, *J =* 8.6 Hz); ^13^C NMR (75 MHz, CDCl_3_) *δ* 39.8, 55.8, 56.9, 75.5, 78.7, 112.3, 114.6, 115.7, 120.3, 134.2, 137.4, 145.0, 149.6.

*General procedure for the synthesis of triazole compounds* ***2a–2h****.*

To a round-bottom flask were added the corresponding azide (1.0 equivalent), alkyne 1 (1.0 equivalent), sodium ascorbate (0.4 equivalent), 2.0 mL of distilled water, and 2.0 mL of ethanol. Subsequently, CuSO_4_∙5H_2_O (0.2 equivalent) was added. The reaction mixture was stirred vigorously at room temperature for 24 to 48 hours. Upon completion of the reaction, as confirmed by TLC, the mixture was washed with a saturated Na_2_CO_3_ solution, and the aqueous phase was extracted with dichloromethane (3 × 20.0 mL). The organic extracts were combined, the organic phase was dried over anhydrous sodium sulfate, filtered, and concentrated under reduced pressure. The resulting material was purified by silica gel column chromatography using a hexane–ethyl acetate mixture (4:1 or 3:2 v v^-1^) as eluent. The structures of compounds **2a–2h** are supported by the following data.

*Synthesis of 4-((4-allyl-2-methoxyphenoxy)methyl)-1-(4-bromophenyl)-1H-1,2,3-triazole (****2a****)* Compound **2a** was obtained as a brown solid in 80% yield (0.157 g, 0.395 mmol), m. p. 124.3─124.8 °C, TLC: R_f_ = 0.30 (hexane**-**ethyl acetate 4:1 v v^-1^). IR (ATR) *v*_max_/cm^-1^: 3128, 3094, 2957, 2924, 2871, 1638, 1590, 1510, 1493, 1462, 1259, 1230, 1138, 1068, 1034, 1025, 987, 926, 824, 804, 749, 650, 605, 517, 453. ^1^H NMR (600 MHz, DMSO-*d*_6_) *δ*: 3.29 (d, 2H, *J* = 6.6 Hz), 3.73 (s, 3H), 5.08─5.01 (m, 2H), 5.16 (s, 2H), 5.96 (ddt_ap_, 1H, *J =* 16.8 Hz, *J =* 9.6 Hz, *J =* 6.6 Hz), 6.70─6.69 (m, 1H), 6.81─6.80 (m, 1H), 7.07 (d, 1H, *J =* 7.8 Hz), 7.80 (d, 2H, *J =* 9.0 Hz), 7.90 (d, 2H, *J =* 9.0 Hz), 8.93 (s, 1H). ^13^C NMR (150 MHz, DMSO-*d*_6_) *δ*: 39.5, 55.8, 62.2, 113.0, 114.6, 116.0, 120.6, 121.8, 122.4, 123.3, 133.2, 133.6, 136.2, 138.2, 144.6, 146.1, 149.5. LC-MS (ESI) *m/z*: calculated for C_19_H_18_BrN_3_O_2_ [M+Na]^+^: 422.05; found: 422.07.

*Synthesis of 4-((4-allyl-2-methoxyphenoxy)methyl)-1-(2-bromophenyl)-1H-1,2,3-triazole (****2b****)* Compound **2b** was obtained as a white solid in 73.0% yield (0.143 g, 0.360 mmol), m. p. 64.6─65.4 °C, TLC: R_f_ = 0.72 (hexane/ethyl acetate 3:2 v v^-1^). IR (ATR) *v*_max_/cm^-1^: 3135, 3072, 2935, 2868, 2838, 1634, 1588, 1508, 1456, 1378, 1256, 1222, 1139, 1021, 1009, 995, 913, 845, 802, 753, 640, 605, 553, 450. ^1^H NMR (600 MHz, DMSO-*d*_6_) *δ* 3.30 (d, 2H, *J =* 6.6 Hz), 3.74 (s, 3H), 5.02─5.09 (m, 2H), 5.17 (s, 2H), 5.96 (ddt, 1H, *J =* 6.6, 10.2, 16.8 Hz), 6.71 (dd, 1H, *J =* 1.8, 8.4 Hz), 6.81─6.82 (m, 1H), 7.08 (d, 1H, *J =* 8.4 Hz), 7.56 (td, 1H, *J =* 1.8, 7.8 Hz), 7.63 (td, 1H, *J =* 1.2, 7.8 Hz), 7.65 (dd, 1H, *J =* 1.8, 7.8 Hz), 7.91 (d, 1H, *J =* 9.0 Hz), 8.62 (s, 1H). ^13^C NMR (150 MHz, DMSO-*d*_6_) *δ* 39.5, 55.8, 62.2, 113.0, 114.9, 115.9, 119.2, 120.6, 127.2, 129.1, 129.3, 132.4, 133.6, 134.0, 136.5, 138.3, 143.3, 146.1, 149.6. LC-MS (ESI) *m/z*, calculated. for C_19_H_18_ BrN_3_NaO_2_ [M + Na]^+^: 422.05, found: 422.05.

*Synthesis of 4-((4-allyl-2-methoxyphenoxy)methyl)-1-(3-bromophenyl)-1H-1,2,3-triazole (****2c****)* Compound **2c** was obtained as a light brown solid in 70.0% yield (0.38 g, 0.346 mmol) ), m. p. 102.2─102.6 °C, TLC: R_f_ = 0.50 (hexane-ethyl acetate 4:1 v v^-1^). IR (ATR) *v*_max_/cm^-1^: 3139, 3076, 2991, 2916, 2849, 1679, 1634, 1588, 1513, 1493, 1460, 1423, 1337, 1258, 1235, 1211, 1136, 1046, 1027, 992, 911, 865, 780, 675, 646, 598, 542, 438. ^1^H NMR (600 MHz, DMSO-*d*_6_) *δ*: 3.28 (d, 2H, *J =* 6.6 Hz), 3.71 (s, 3H), 5.99─5.06 (m, 2H), 5.14 (s, 2H), 5.88─5.95 (m, 1H), 6.68 (d, 1H, *J =* 8.4 Hz), 6.78 (s, 1H), 7.04 (d, 1H, *J =* 8.4 Hz), 7.54 (t, 1H, *J =* 8.1 Hz), 7.68 (d, 1H, *J =* 8.4 Hz), 7.94 (d, 1H, *J =* 8.4 Hz), 8.15 (s, 1H), 8.96 (s, 1H). ^13^C NMR (150 MHz, DMSO-*d*_6_) *δ*: 39.5, 55.8, 62.2, 113.0, 114.7, 116.0, 119.5, 120.6, 122.8, 123.1, 123.5, 131.9, 132.3, 133.6, 138.1, 138.3, 144.6, 146.1, 149.6. LC-MS (ESI) *m/z*: calculated for C_19_H_18_BrN_3_O_2_ [M+Na]^+^: 422.05; found: 422.13.

*Synthesis of 4-((4-allyl-2-methoxyphenoxy)methyl)-1-(4-chlorophenyl)-1H-1,2,3-triazole (****2d****)* Compound **2d** was obtained as a white solid in 83.0% yield (0.145 g, 0.410 mmol), m. p. 124.8─125.3 °C, TLC: R_f_ = 0.48 (hexane-ethyl acetate 4:1 v v^-1^). IR (ATR) *v*_max_/cm^-1^: 3132, 3098, 2998, 2924, 2875, 2834, 1638, 1590, 1510, 1497, 1463, 1404, 1337, 1260, 1231, 1218, 1140, 1090, 1036, 1026, 992, 926, 826, 751, 648, 605, 520, 470. ^1^H NMR (600 MHz, DMSO-*d*_6_) *δ*: 3.29 (d, 2H, *J =* 6.6 Hz), 3.73 (s, 3H), 5.01─5.08 (m, 2H), 5.16 (s, 2H), 5.96 (ddt_ap_, 1H, *J =* 16.8 Hz, *J =* 10.2 Hz, *J =* 6.6 Hz), 6.69─6.70 (m, 1H), 6.80─6.81 (m, 1H), 7.07 (d, 1H, *J =* 7.8 Hz), 7.67 (d, 2H, *J =* 9.0 Hz), 7.95 (d, 2H, *J =* 9.0 Hz), 8.94 (s, 1H). ^13^C NMR (150 MHz, DMSO-*d*_6_) *δ*: 39.5, 55.8, 62.2, 113.0, 114.7, 116.0, 120.6, 122.2, 123.4, 130.3, 133.4, 133.6, 135.8, 138.3, 144.6, 146.1, 149.5. LC-MS (ESI) *m/z*: calculated for C_19_H_18_ClN_3_O_2_ [M+Na]^+^: 378.10; found: 378.09.

*Synthesis of 4-((4-allyl-2-methoxyphenoxy)methyl)-1-(3-chlorophenyl)-1H-1,2,3-triazole (****2e****)* Compound **2e** was obtained as a white solid in 76.0% yield (0.133 g, 0.375 mmol), m. p. 104.2─104.8 °C, TLC: R_f_ = 0.46 (hexane-ethyl acetate 4:1 v v^-1^). IR (ATR) *v*_max_/cm^-1^: 3143, 3076, 2991, 2912, 2831, 1638, 1594, 1514, 1496, 1460, 1423, 1259, 1235, 1211, 1136, 1030, 993, 911, 866, 781, 753, 781, 676, 646, 598, 546, 442; ^1^H NMR (600 MHz, DMSO-*d*_6_) *δ*: 3.30 (d, 2H, *J =* 7.2 Hz), 3.73 (s, 3H), 5.01─5.08 (m, 2H), 5.16 (s, 2H), 5.96 (ddt_ap_, 1H, *J =* 16.8 Hz, *J =* 9.6 Hz, *J =* 6.6 Hz), 6.70 (dd, 1H, *J =* 2.4 Hz, *J =* 8.4 Hz), 6.80─6.81 ( m, 1H), 7.06 (d, 1H, *J =* 8.4 Hz), 7.56─7.57 (m, 1H), 7.63 (t, 1H, *J =* 8.1 Hz), 7.93─7.94 (m, 1H), 8.05─8.06 (m, 1H), 8.99 (s, 1H). ^13^C NMR (150 MHz, DMSO-*d*_6_) *δ*: 41.7, 58.0, 64.4, 115.2, 116.9, 118.1, 121.3, 122.5, 122.7, 125.7, 131.1, 134.2, 135.8, 136.8, 140.2, 140.4, 146.8, 148.2, 151.7. LC-MS (ESI) *m/z*: calculated for C_19_H_18_ClN_3_O_2_ [M+Na]^+^: 378.10; found: 378.16.

*Synthesis of 4-((4-allyl-2-methoxyphenoxy)methyl)-1-(2-chlorophenyl)-1H-1,2,3-triazole (****2f****)* Compound **2f** was obtained as a pasty white solid in 68.0% yield (0.120 g, 0.336 mmol), m. p. 56.1─56.8 ºC, TLC: R_f_ = 0.54 (hexane-ethyl acetate 3:2 v v^-1^). IR (ATR) *v*_max_/cm^-1^: 3143, 3076, 3002, 2935, 2871, 2834, 1716, 1638, 1590, 1508, 1496, 1459, 1419, 1257, 1224, 1138, 1035, 1017, 911, 847, 804, 756, 647, 602, 546, 459. ^1^H NMR (400 MHz, CDCl_3_) *δ*: 3.34 (d, 2H, *J* = 6.4 Hz), 3.86 (s, 3H), 5.06─5.10 (m, 2H), 5.38 (s, 2H), 5.90─6.00 (m, 1H), 6.71─6.73 (m, 2H), 7.01 (d, 1H, *J* = 8.0 Hz), 7.44─7.46 (m, 2H), 7.56─7.62 (m, 2H), 8.08 (s, 1H). ^13^C NMR (100 MHz, CDCl_3_) *δ*: 40.0, 56.0, 63.5, 112.6, 115.2, 115.9, 120.7, 125.3, 128.0, 128.1, 131.0, 131.0, 134.3, 137.7, 144.4, 146.0, 149.9. LC-MS (ESI) *m/z*: calculado para C_19_H_18_ClN_3_O_2_ [M+Na]^+^: 378.10; encontrado: 378.10.

*Synthesis of 4-((4-allyl-2-methoxyphenoxy)methyl)-1-phenyl-1H-1,2,3-triazole (****2g****)*

Compound **2g** was obtained as a white solid in 62% yield (0.098 g, 0.305 mmol), m. p. 78.3─78.9 °C, TLC: R_f_ = 0.32 (hexane-ethyl acetate 4:1 v v^-1^). IR (ATR) *v*_max_/cm^-1^: 3135, 3080, 2994, 2935, 2905, 2827, 1638, 1592, 1501, 1463, 1423, 1385, 1330, 1258, 1222, 1139, 1033, 1001, 983, 913, 834, 762, 689, 650, 598, 518, 464. ^1^H NMR (300 MHz, CDCl_3_) *δ*: 3.33 (d, 2H, *J* = 6.9 Hz), 3.86 (s, 3H), 5.04─5.10 (m, 2H), 5.35 (s, 2H), 5.98 (ddt_ap_, 1H, *J =* 16.8 Hz, *J =* 10.2 Hz, *J =* 6.9 Hz), 6.70─6.73 (m, 2H), 7.00 (d, 1H, *J =* 7.8 Hz), 7.40─7.54 (m, 3H), 7.71 (d, 2H, *J =* 8.1 Hz), 8.07 (s, 1H). ^13^C NMR (75 MHz, CDCl_3_) *δ*: 39.8, 55.8, 63.3, 112.3, 114.4, 115.7, 120.5, 120.5, 121.0, 128.8, 129.7, 133.9, 136.9, 137.4, 145.2, 145.8, 149.5. LC-MS (ESI) *m/z*: calculated for C_19_H_18_N_3_O_2_ [M+Na]^+^: 344.14; found: 344.22.

*Synthesis of 4-((4-allyl-2-methoxyphenoxy)methyl)-1-(4-fluorophenyl)-1H-1,2,3-triazole (****2h****)* Compound **2h** was obtained as a white solid in 69% yield (0.073 g, 0.215 mmol), m. p. 80.4─81.2 °C, TLC: R_f_ = 0.46 (hexane-ethyl acetate 4:1 v v^-1^). IR (ATR) *v*_max_/cm^-1^: 3124, 3065, 3005, 2935, 2879, 2831, 1638, 1592, 1510, 1463, 1420, 1378, 1222, 1138, 1051, 1031, 1003, 995, 911, 836, 799, 761, 698, 646, 602, 518, 479. ^1^H NMR (600 MHz, DMSO-*d*_6_) *δ*: 3.30 (d, 2H, *J =* 7.2 Hz), 3.73 (s, 3H), 5.01─5.08 (m, 2H), 5.16 (s, 2H), 5.96 (ddt_ap_, 1H, *J =* 16.8 Hz, *J =* 10.2 Hz, *J =* 7.2 Hz), 6.70 (dd_ap_, 1H, *J_1_ =* 1.8 and *J_2_ =* 7.8 Hz), 6.80─6.81 (m, 1H), 7.07 (d, 1H, *J =* 8.4 Hz), 7.46 (t, 2H, *J =* 8.7 Hz), 7.93 (dd, 2H, *J_1_ =* 4.8 and *J_2_ =* 9.0 Hz), 8.89 (s, 1H). ^13^C NMR (150 MHz, DMSO-*d*_6_) *δ*: 39.5, 55.8, 62.2, 113.0, 114.6, 116.0, 117,1 (d, *J* = 9.0 Hz), 120.6, 122.9 (d, *J* = 9.0 Hz), 123.5, 133.5, 133.6, 138.3, 144.5, 146.1, 149.5, 162.1 (d, *J* = 244.5 Hz). LC-MS (ESI) *m/z*: calculado para C_19_H_18_FN_3_O_2_ [M+Na]^+^: 362.13; encontrado: 362.15.

**3. IR, NMR (^1^H AND ^13^C) AND LC-MS/MS SPECTRA OF COMPOUNDS 1 AND TRIAZOLES 2a─2h**

**
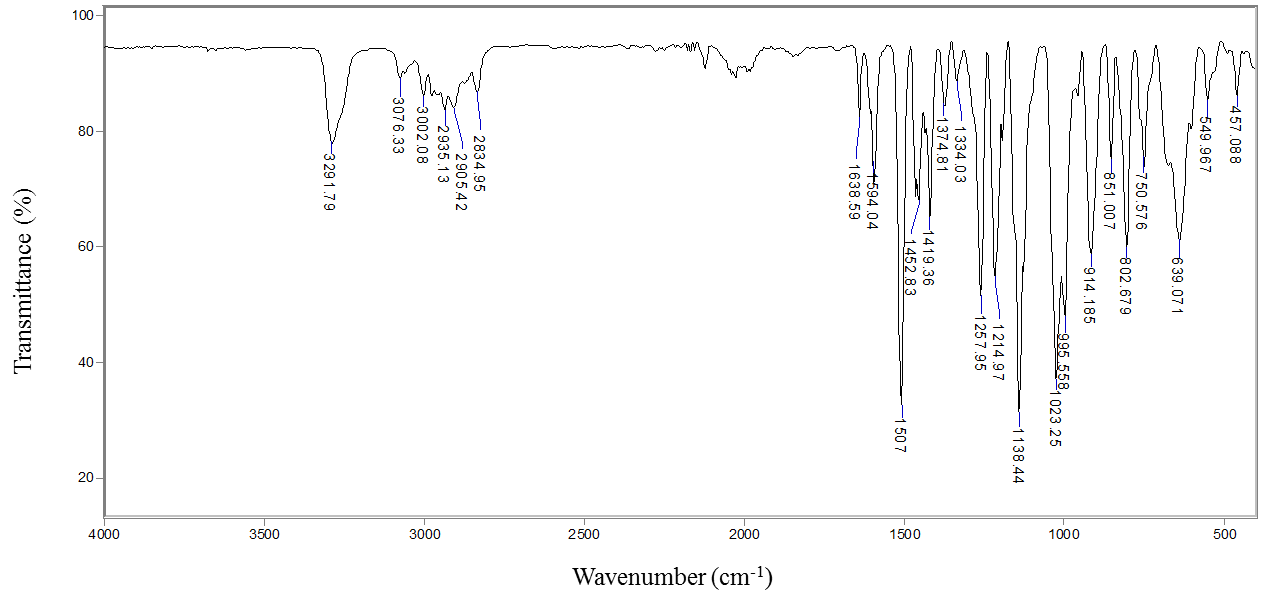
**

**Figure.** **S1.** FTIR spectrum (ATR) of 4-allyl-2-methoxy-1-(prop-2-yn-1-yloxy)benzene **1**.

**Figure.** **S2.** ^1^H NMR spectrum (300 MHz, CDCl_3_) of 4-allyl-2-methoxy-1-(prop-2-yn-1-yloxy)benzene **1**.

*δ*

**Figure.** **S3.** ^13^C NMR spectrum (75 MHz, CDCl_3_) of 4-allyl-2-methoxy-1-(prop-2-yn-1-yloxy)benzene **1**.

**
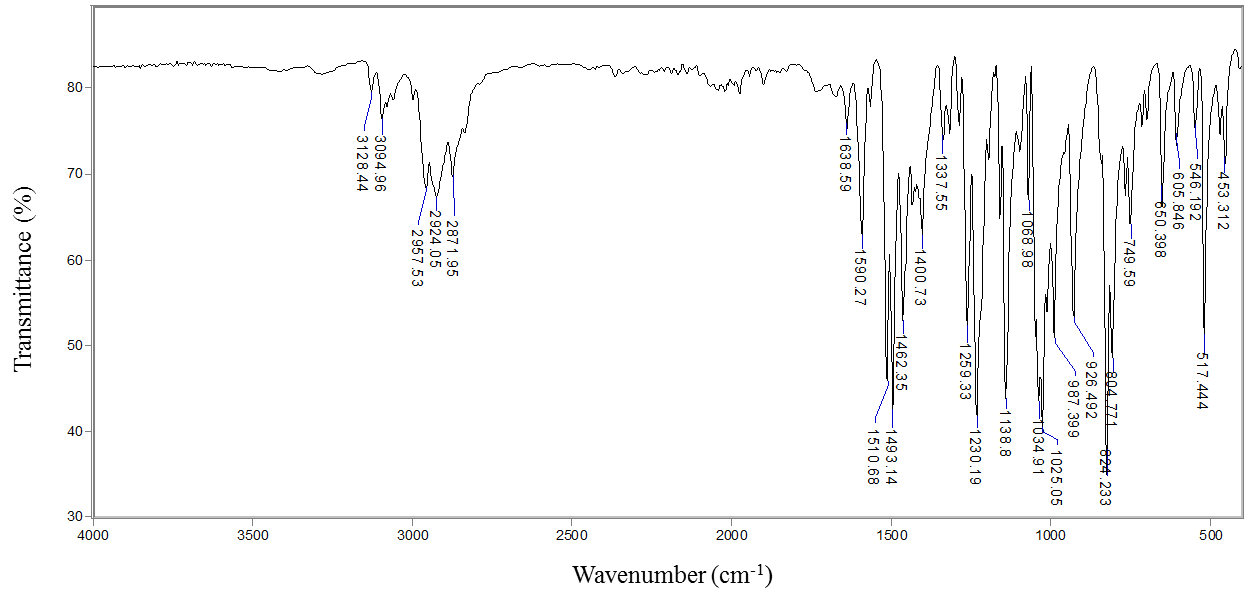
**

**Figure.** **S4.** FTIR spectrum (ATR) of 4-((4-allyl-2-methoxyphenoxy)methyl)-1-(4-bromophenyl)-1*H*-1,2,3-triazole **2a**.

**Figure.** **S5.** ^1^H NMR spectrum (600 MHz, DMSO-*d_6_*) of 4-((4-allyl-2-methoxyphenoxy)methyl)-1-(4-bromophenyl)-1*H*-1,2,3-triazole **2a**.

**Figure.** **S6.** ^13^C NMR spectrum (150 MHz, DMSO-*d_6_*) of 4-((4-allyl-2-methoxyphenoxy)methyl)-1-(4-bromophenyl)-1*H*-1,2,3-triazole **2a**.

**
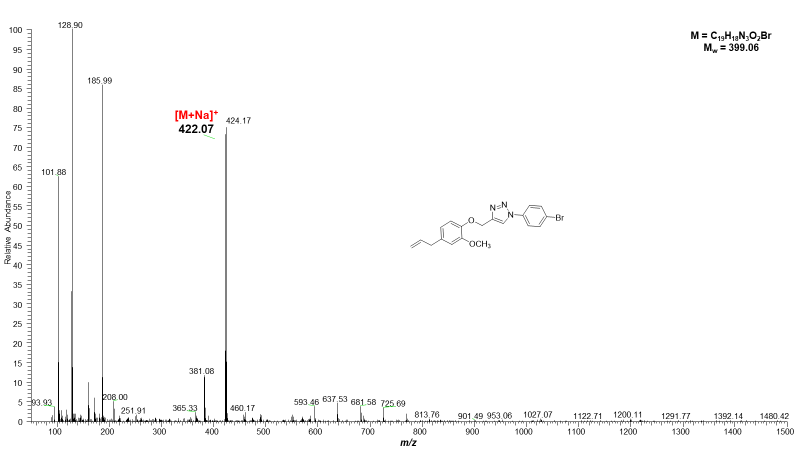
**

**Figure.** **S7.** LC-MS spectrum of 4-((4-allyl-2-methoxyphenoxy)methyl)-1-(4-bromophenyl)-1*H*-1,2,3-triazole **2a**.

**
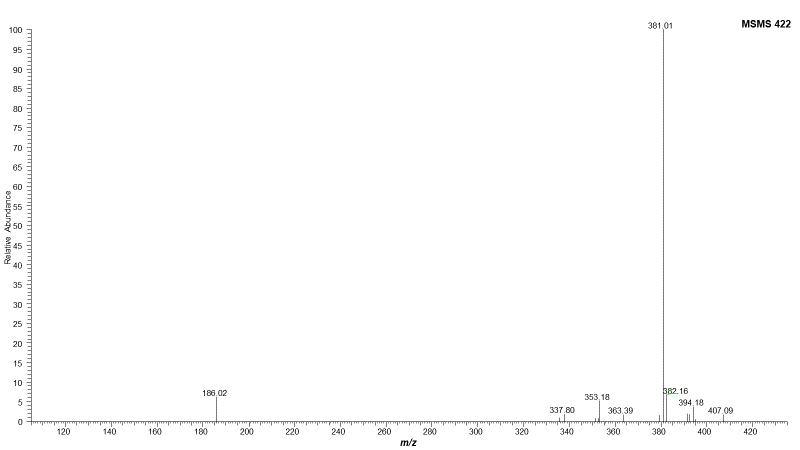
**

**Figure.** **S8.** MS-MS spectrum of 4-((4-allyl-2-methoxyphenoxy)methyl)-1-(4-bromophenyl)-1*H*-1,2,3-triazole **2a**.

**
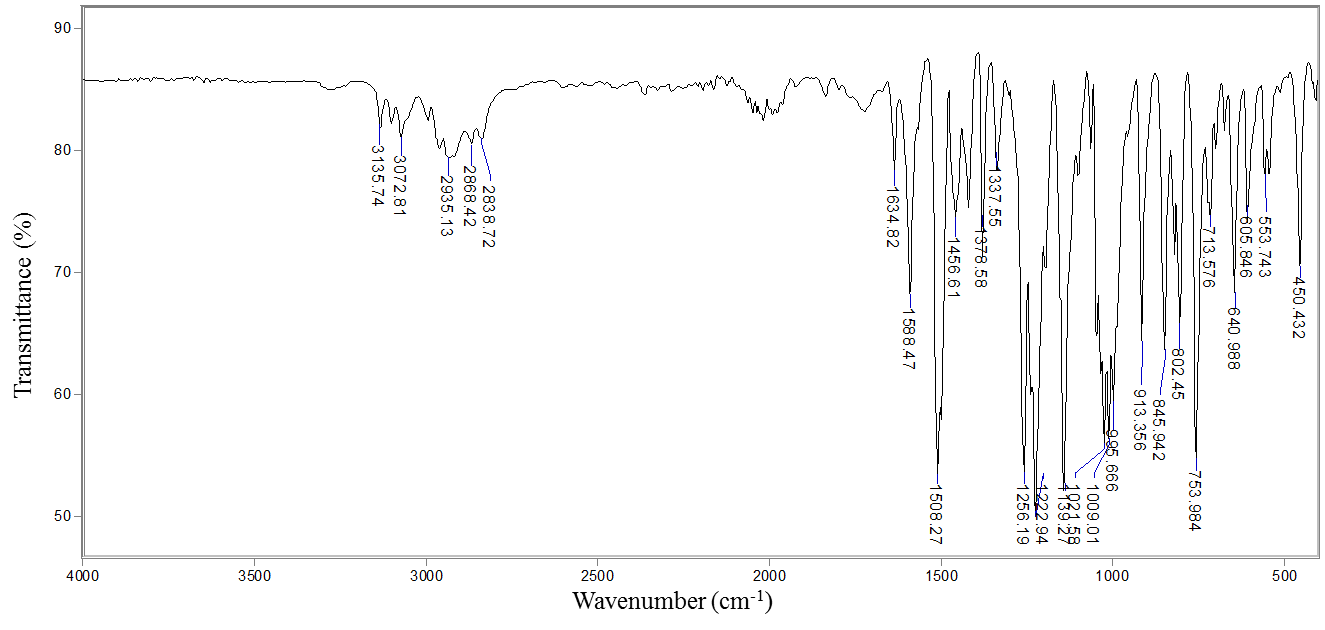
**

**Figure.** **S9.** FTIR spectrum (ATR) of 4-((4-allyl-2-methoxyphenoxy)methyl)-1-(2-bromophenyl)-1*H*-1,2,3-triazole **2b**.

**Figure.** **S10.** ^1^H NMR spectrum (600 MHz, DMSO-*d_6_*) of 4-((4-allyl-2-methoxyphenoxy)methyl)-1-(2-bromophenyl)-1*H*-1,2,3-triazole **2b**.

*δ*

**Figure.** **S11.** ^13^C NMR spectrum (150 MHz, DMSO-*d_6_*) of 4-((4-allyl-2-methoxyphenoxy)methyl)-1-(2-bromophenyl)-1*H*-1,2,3-triazole **2b**.

**
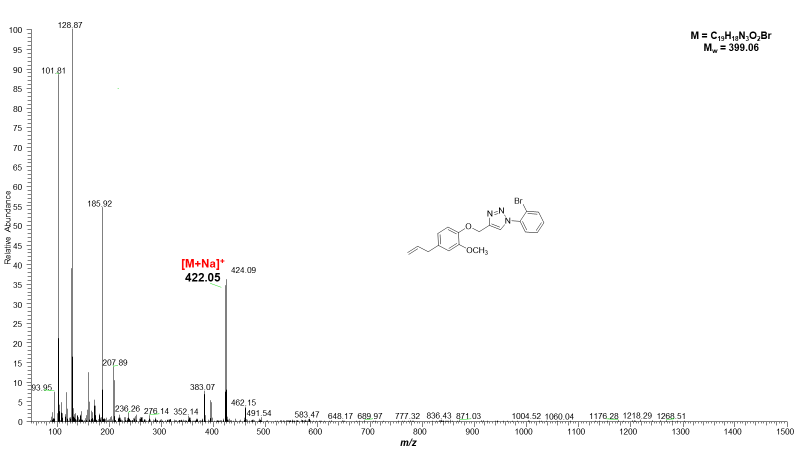
**

**Figure.** **S12.** LC-MS spectrum of 4-((4-allyl-2-methoxyphenoxy)methyl)-1-(2-bromophenyl)-1*H*-1,2,3-triazole **2b**.

**
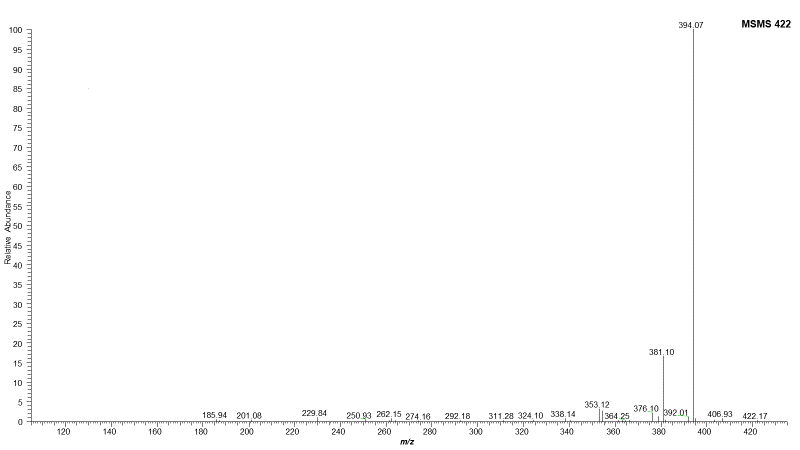
**

**Figure.** **S13.** MS-MS spectrum of 4-((4-allyl-2-methoxyphenoxy)methyl)-1-(2-bromophenyl)-1*H*-1,2,3-triazole **2b**.

**
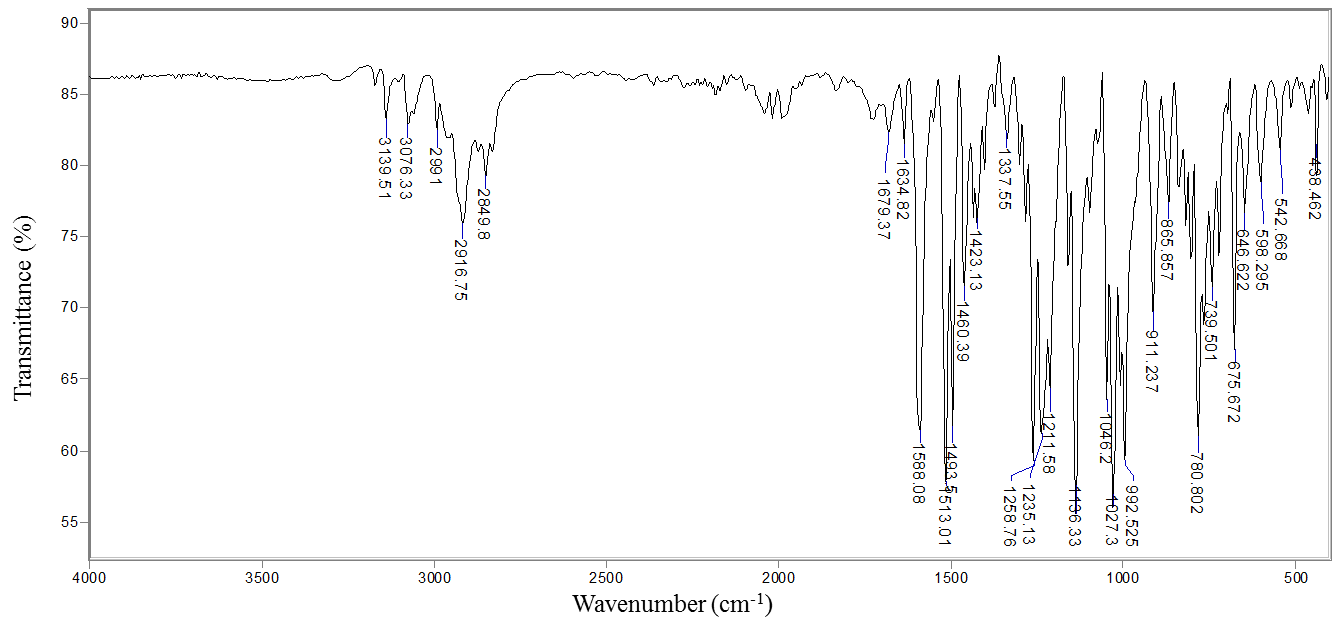
**

**Fig.** **S14.** FTIR spectrum (ATR) of 4-((4-allyl-2-methoxyphenoxy)methyl)-1-(3-bromophenyl)-1*H*-1,2,3-triazole **2c**.

**Figure.** **S15.** ^1^H NMR spectrum (600 MHz, DMSO-*d_6_*) of 4-((4-allyl-2-methoxyphenoxy)methyl)-1-(3-bromophenyl)-1*H*-1,2,3-triazole **2c**.

**Figure.** **S16.** ^13^C NMR spectrum (150 MHz, DMSO-*d_6_*) of 4-((4-allyl-2-methoxyphenoxy)methyl)-1-(3-bromophenyl)-1*H*-1,2,3-triazole **2c**.

**
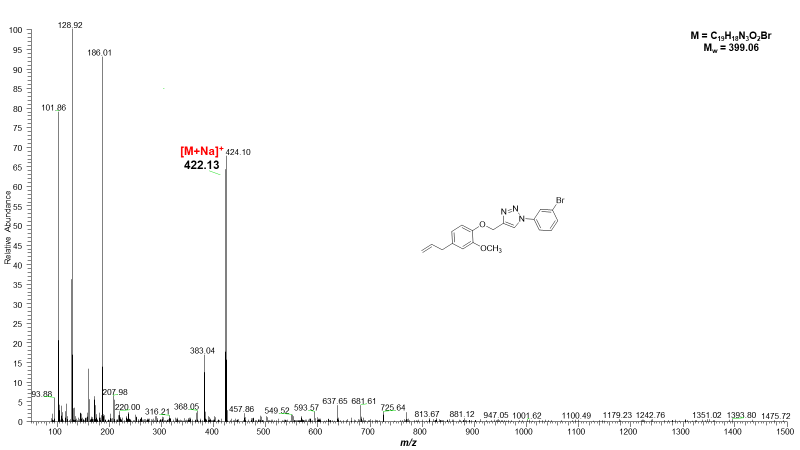
**

**Figure.** **S17.** LC-MS spectrum of 4-((4-allyl-2-methoxyphenoxy)methyl)-1-(3-bromophenyl)-1*H*-1,2,3-triazole **2c**.

**
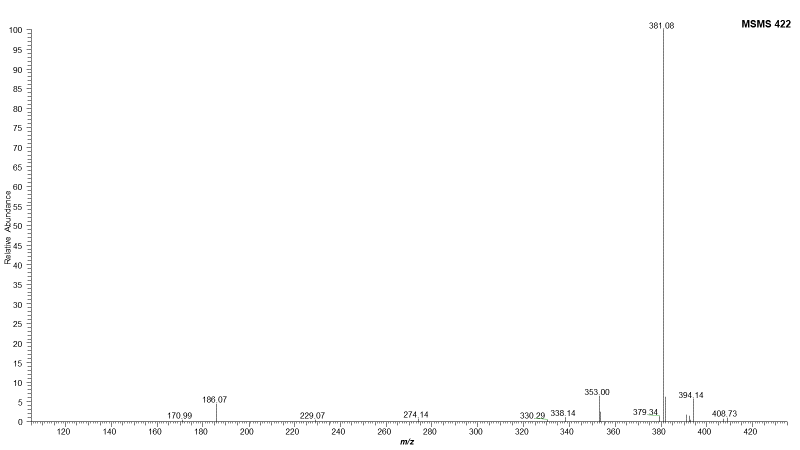
**

**Figure.** **S18.** MS-MS spectrum of 4-((4-allyl-2-methoxyphenoxy)methyl)-1-(3-bromophenyl)-1*H*-1,2,3-triazole **2c**.

**
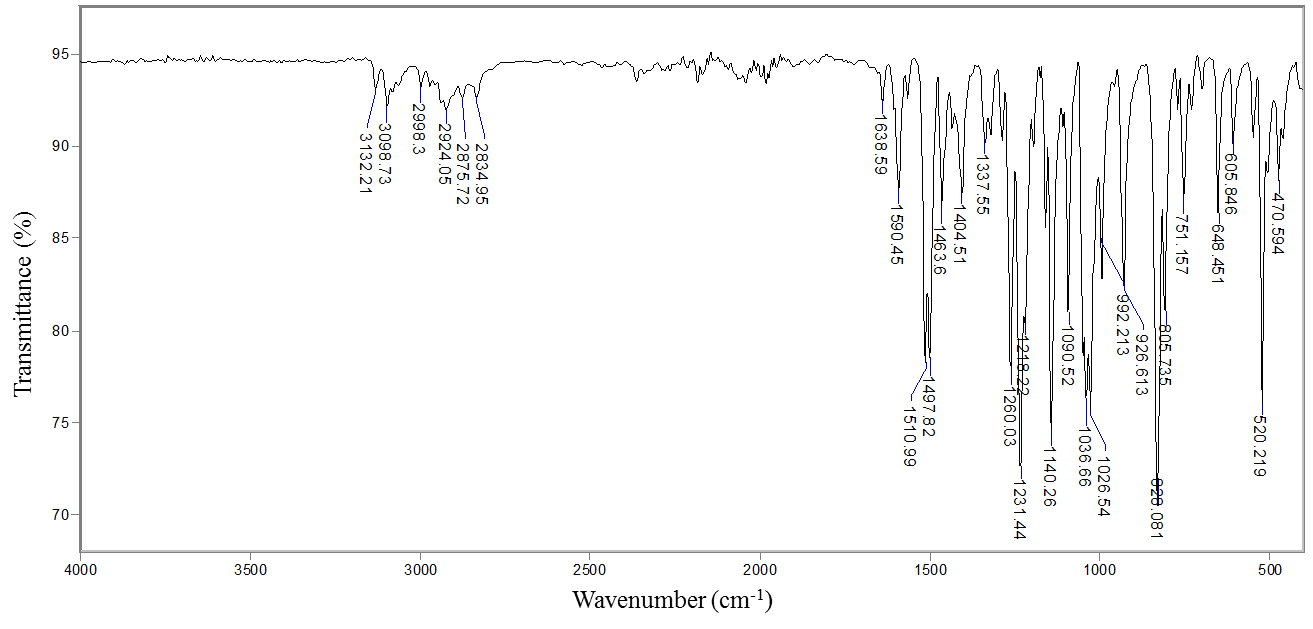
**

**Figure.** **S19.** FTIR spectrum (ATR) of 4-((4-allyl-2-methoxyphenoxy)methyl)-1-(4-chlorophenyl)-1*H*-1,2,3-triazole **2d**.

**Figure.** **S20.** ^1^H NMR spectrum (600 MHz, DMSO-*d_6_*) of 4-((4-allyl-2-methoxyphenoxy)methyl)-1-(4-chlorophenyl)-1*H*-1,2,3-triazole **2d**.

**Figure.** **S21.** ^13^C NMR spectrum (150 MHz, DMSO-*d_6_*) of 4-((4-allyl-2-methoxyphenoxy)methyl)-1-(4-chlorophenyl)-1*H*-1,2,3-triazole **2d**.


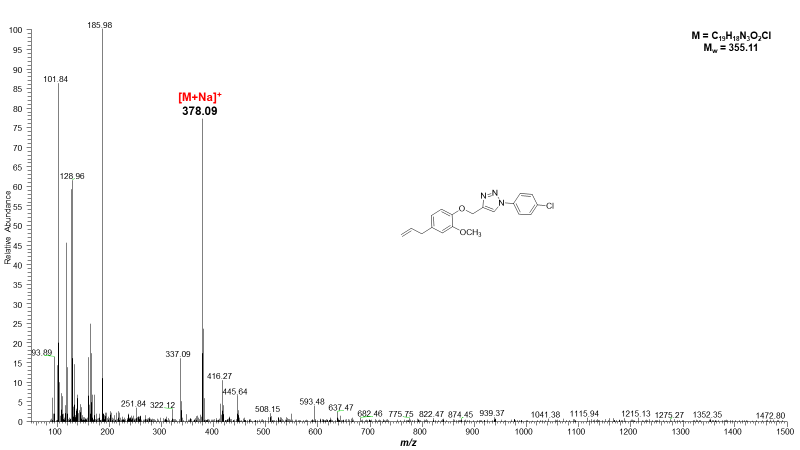


**Figure.** **S22.** LC-MS spectrum of 4-((4-allyl-2-methoxyphenoxy)methyl)-1-(4-chlorophenyl)-1*H*-1,2,3-triazole **2d**.

**
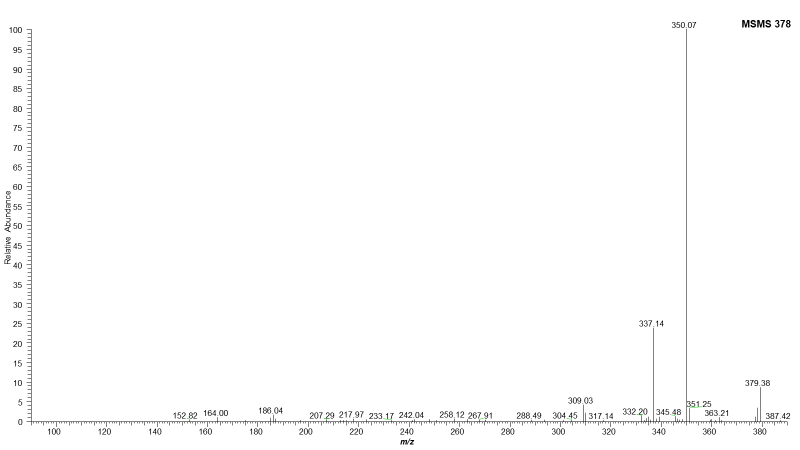
**

**Figure.** **S23.** MS-MS spectrum of 4-((4-allyl-2-methoxyphenoxy)methyl)-1-(4-chlorophenyl)-1*H*-1,2,3-triazole **2d**.

**
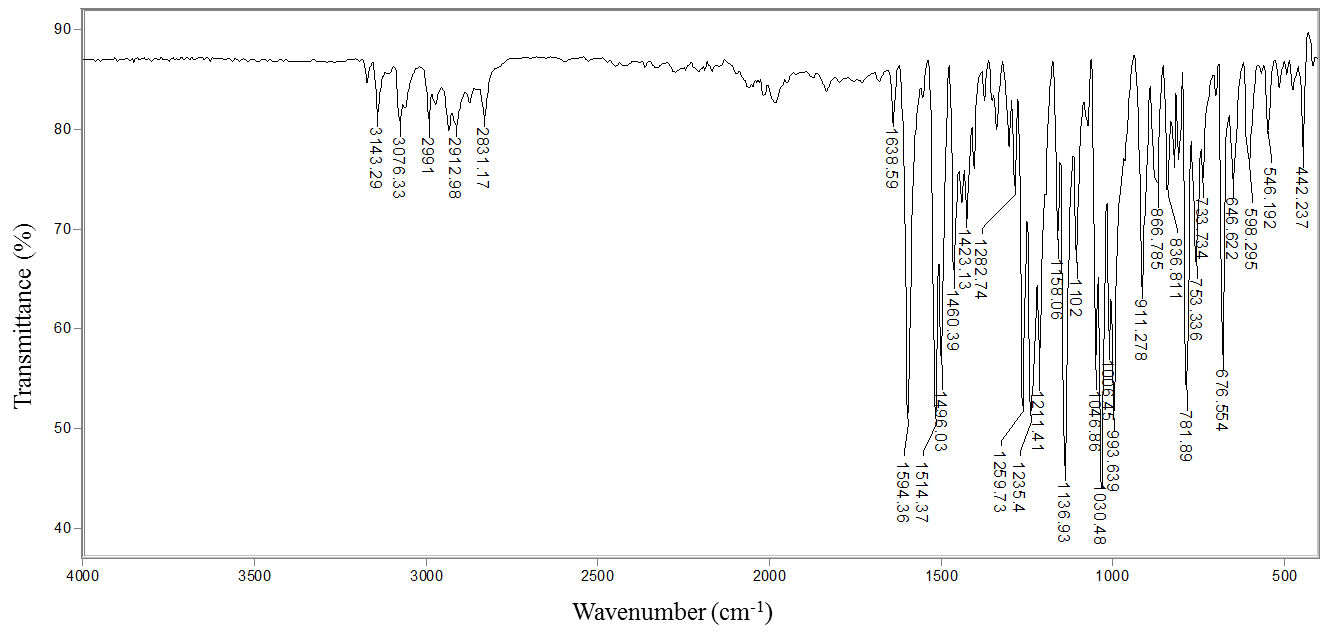
**

**Figure.** **S24.** FTIR spectrum (ATR) of 4-((4-allyl-2-methoxyphenoxy)methyl)-1-(3-chlorophenyl)-1*H*-1,2,3-triazole **2e**.

**Figure.** **S25.** ^1^H NMR spectrum (600 MHz, DMSO-*d_6_*) of 4-((4-allyl-2-methoxyphenoxy)methyl)-1-(3-chlorophenyl)-1*H*-1,2,3-triazole **2e**.

**Figure.** **S26.** ^13^C NMR spectrum (150 MHz, DMSO-*d_6_*) of 4-((4-allyl-2-methoxyphenoxy)methyl)-1-(3-chlorophenyl)-1*H*-1,2,3-triazole **2e**.

**
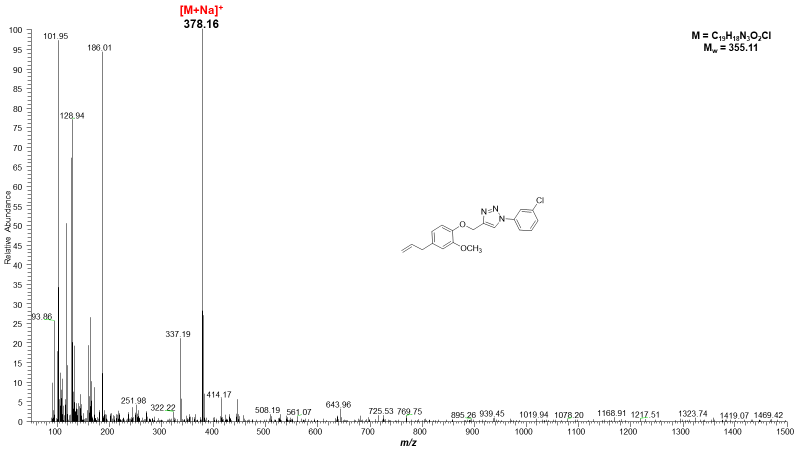
**

**Figure.** **S27.** LC-MS spectrum of 4-((4-allyl-2-methoxyphenoxy)methyl)-1-(3-chlorophenyl)-1*H*-1,2,3-triazole **2e**.

**
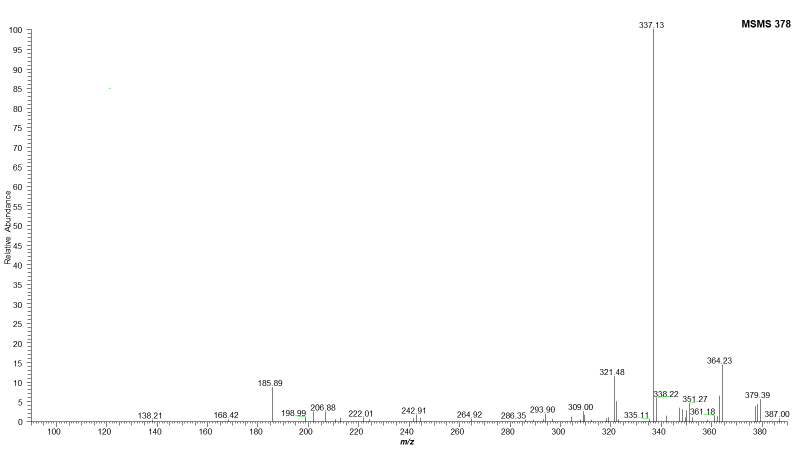
**

**Figure.** **S28.** MS-MS spectrum of 4-((4-allyl-2-methoxyphenoxy)methyl)-1-(3-chlorophenyl)-1*H*-1,2,3-triazole **2e**.

**
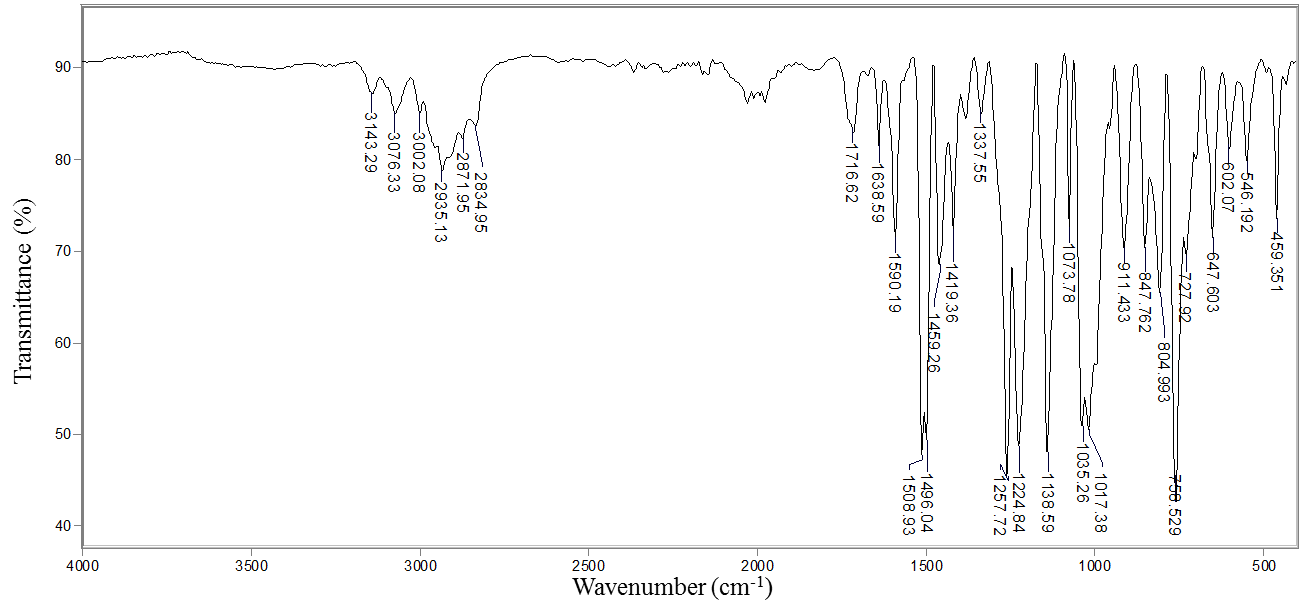
**

**Figure.** **S29.** FTIR spectrum (ATR) of 4-((4-allyl-2-methoxyphenoxy)methyl)-1-(2-chlorophenyl)-1*H*-1,2,3-triazole **2f**.

**Figure.** **S30.** ^1^H NMR spectrum (400 MHz, CDCl_3_) of 4-((4-allyl-2-methoxyphenoxy)methyl)-1-(2-chlorophenyl)-1*H*-1,2,3-triazole **2f**.

**Figure.** **S31.** ^13^C NMR spectrum (100 MHz, CDCl_3_) of 4-((4-allyl-2-methoxyphenoxy)methyl)-1-(2-chlorophenyl)-1*H*-1,2,3-triazole **2f**.


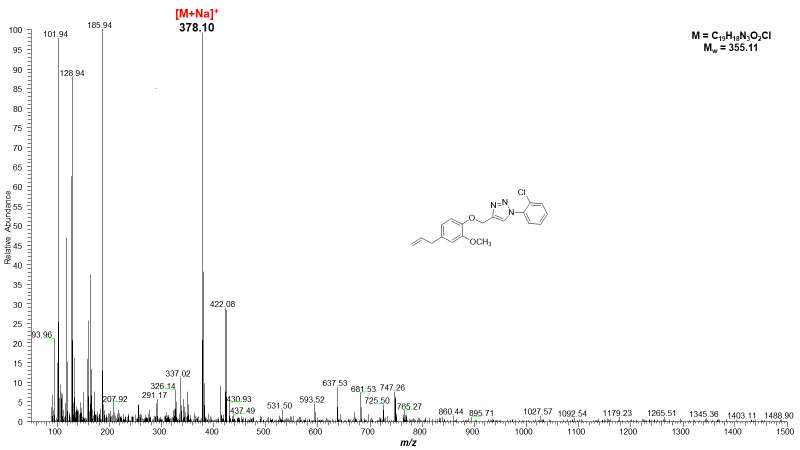


**Figure.** **S32.** LC-MS spectrum of 4-((4-allyl-2-methoxyphenoxy)methyl)-1-(2-chlorophenyl)-1*H*-1,2,3-triazole **2f**.


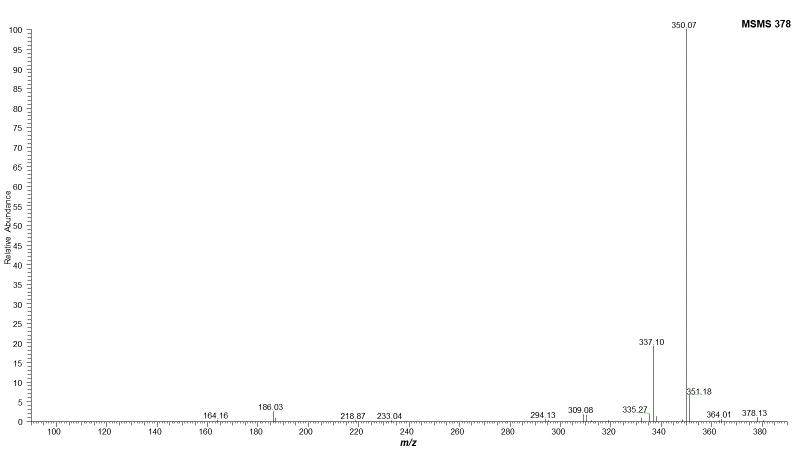


**Figure.** **S33.** MS-MS spectrum of 4-((4-allyl-2-methoxyphenoxy)methyl)-1-(2-chlorophenyl)-1*H*-1,2,3-triazole **2f**.

**
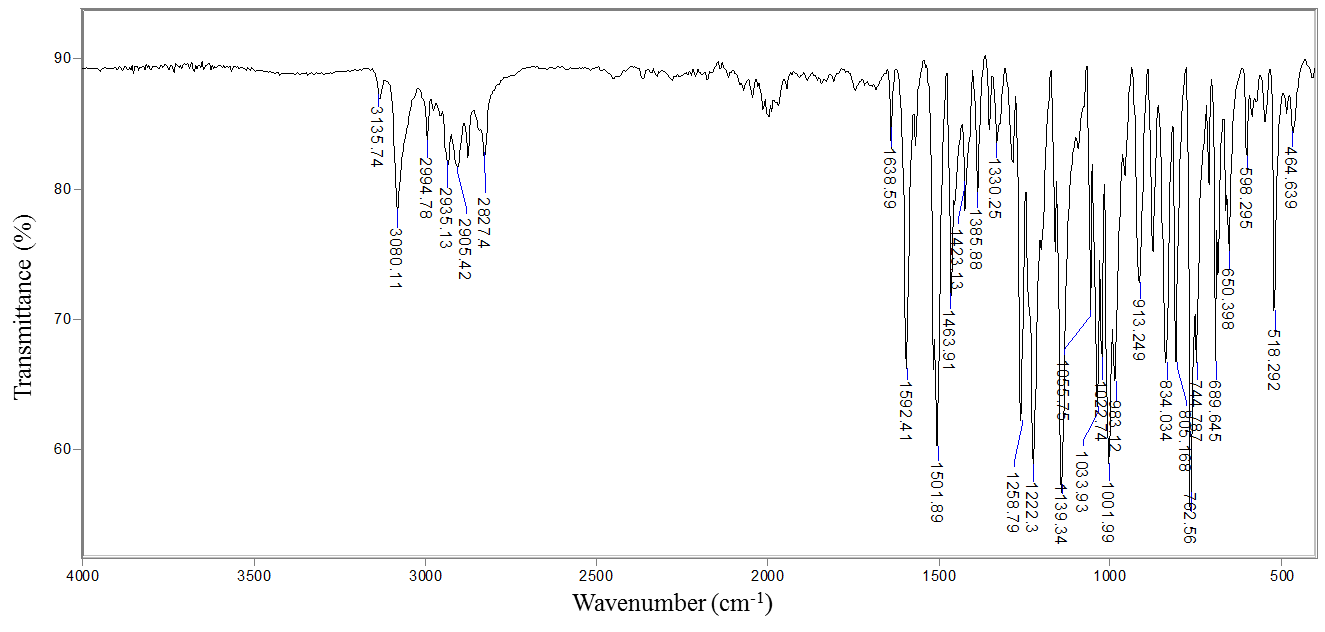
**

**Figure.** **S34.** FTIR spectrum (ATR) of 4-((4-allyl-2-methoxyphenoxy)methyl)-1-phenyl-1*H*-1,2,3-triazole **2g**.

**Figure.** **S35.** ^1^H NMR spectrum (300 MHz, CDCl_3_) of 4-((4-allyl-2-methoxyphenoxy)methyl)-1-phenyl-1*H*-1,2,3-triazole **2g**.

**Figure.** **S36.** ^13^C NMR spectrum (75 MHz, CDCl_3_) of 4-((4-allyl-2-methoxyphenoxy)methyl)-1-phenyl-1*H*-1,2,3-triazole **2i**.


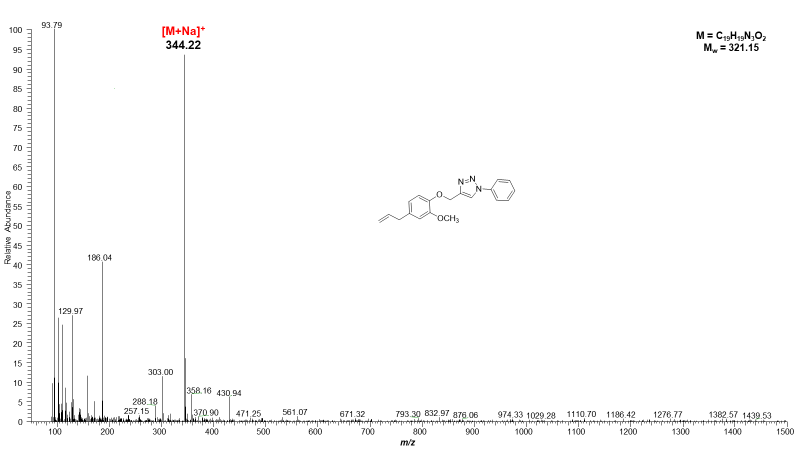


**Figure.** **S37.** LC-MS spectrum of 4-((4-allyl-2-methoxyphenoxy)methyl)-1-phenyl-1*H*-1,2,3-triazole **2g**.


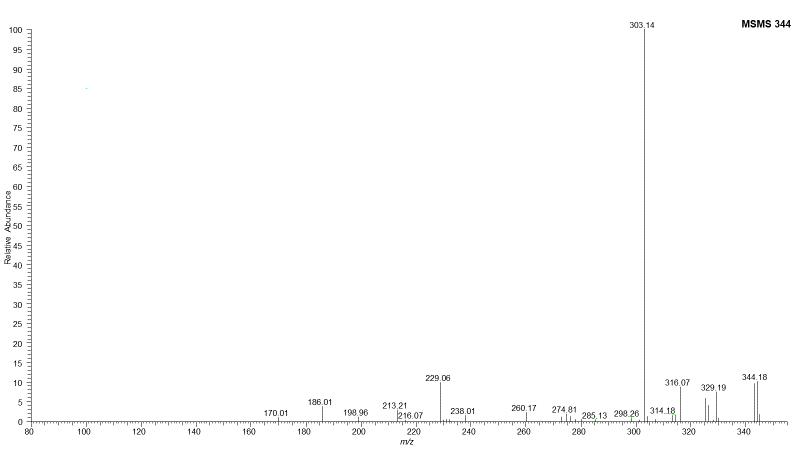


**Figure.** **S38.** MS-MS spectrum of 4-((4-allyl-2-methoxyphenoxy)methyl)-1-phenyl-1*H*-1,2,3-triazole **2g**.

**
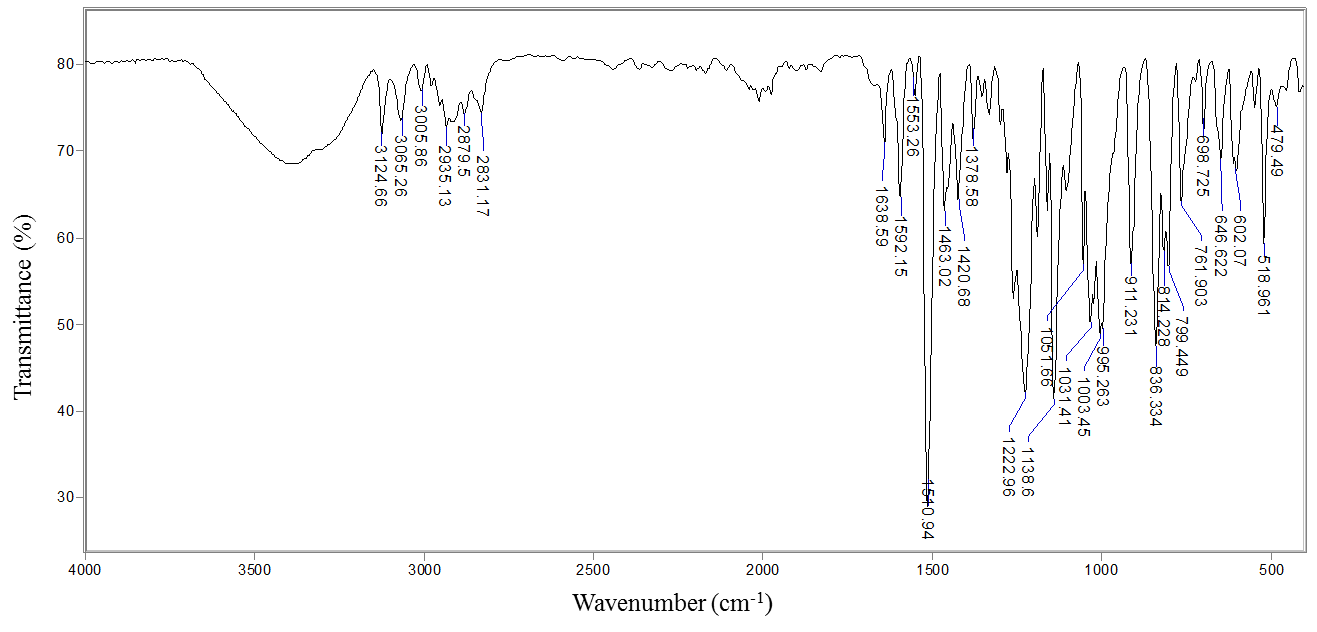
**

**Figure.** **S39.** FTIR spectrum (ATR) of 4-((4-allyl-2-methoxyphenoxy)methyl)-1-(4-fluorophenyl)-1*H*-1,2,3-triazole **2h**.

**Figure.** **S40.** ^1^H NMR spectrum (600 MHz, DMSO-*d_6_*) of 4-((4-allyl-2-methoxyphenoxy)methyl)-1-(4-fluorophenyl)-1*H*-1,2,3-triazole **2h**.

**Figure.** **S41.** ^13^C NMR spectrum (150 MHz, DMSO-*d*_6_) of 4-((4-allyl-2-methoxyphenoxy)methyl)-1-(4-fluorophenyl)-1*H*-1,2,3-triazole **2h**.


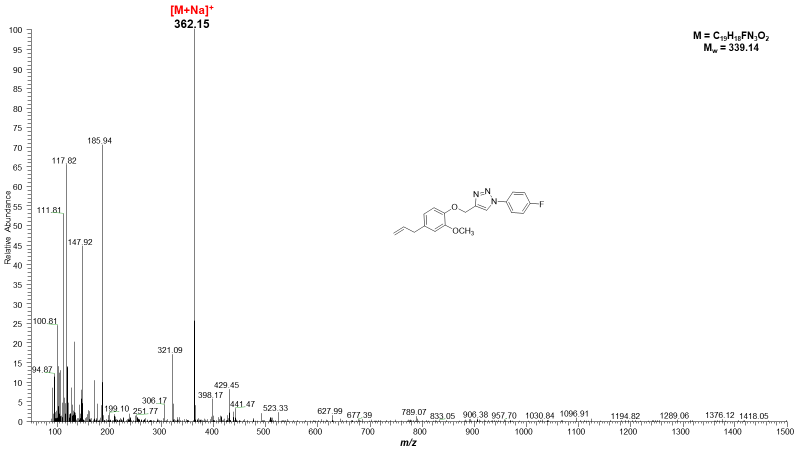


**Figure.** **S42.** LC-MS spectrum of 4-((4-allyl-2-methoxyphenoxy)methyl)-1-(4-fluorophenyl)-1*H*-1,2,3-triazole **2h**.

**
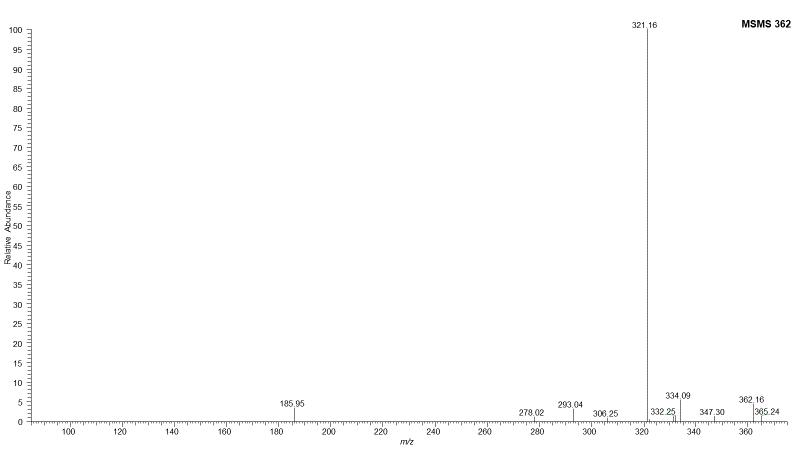
**

**Figure.** **S43.** MS-MS spectrum of 4-((4-allyl-2-methoxyphenoxy)methyl)-1-(4-fluorophenyl)-1*H*-1,2,3-triazole **2h**.

**4. COMPUTATIONAL ANALYSIS DATA**


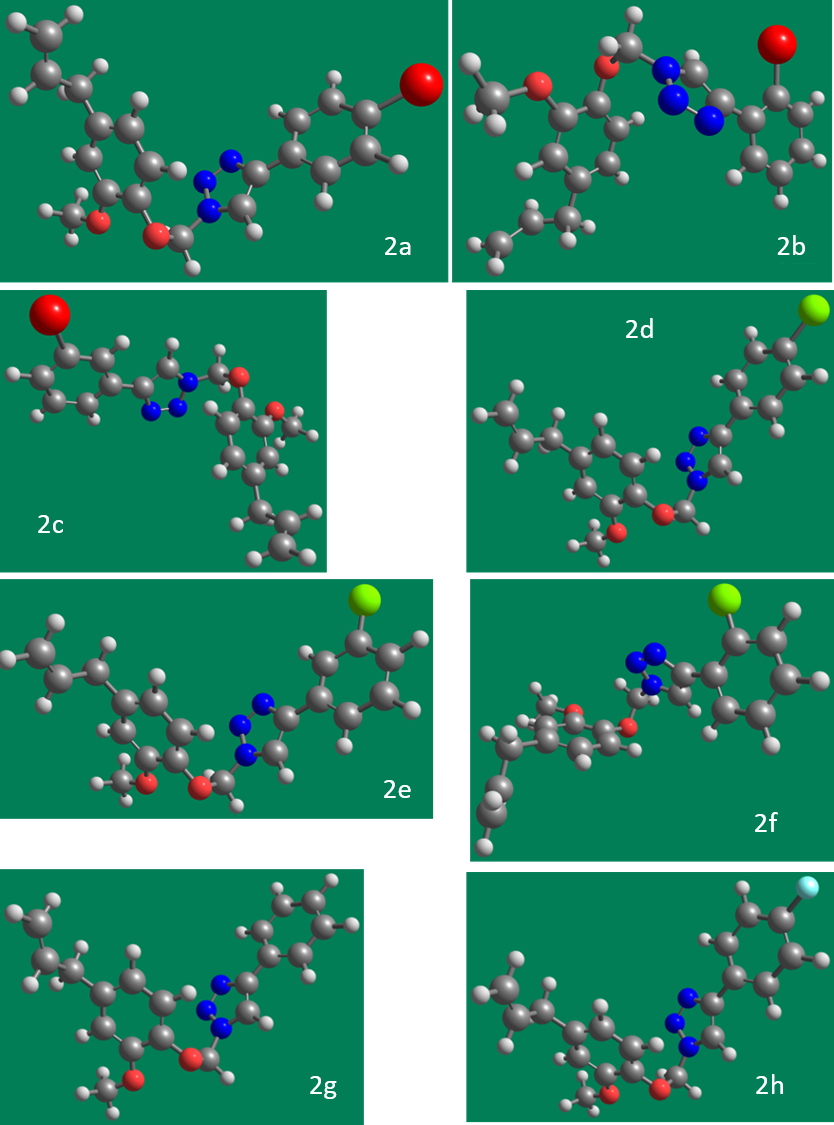


**Figure S441.** 3D structure of the studied compounds from ab initio calculations.
